# Visible light responsive heterophase Titania monoliths for the fast and efficient photocatalytic decontamination of organic pollutants

**DOI:** 10.1038/s41598-024-79285-3

**Published:** 2024-11-10

**Authors:** Denna Babu, Dhivya Jagadeesan, T. V. L. Thejaswini, Akhila Maheswari Mohan, Prabhakaran Deivasigamani

**Affiliations:** Department of Chemistry, School of Advanced Sciences, Vellore Institute of Technology (VIT), Vellore, Tamil Nadu 632014 India

**Keywords:** Undoped Titania, Heterophase, Mesoporous Monolith, Visible light, Photocatalysis, Chemistry, Materials science, Nanoscience and technology

## Abstract

The article reports the synthesis of an ordered mesoporous network of heterophase TiO_2_ monoliths as a visible light-responsive photocatalyst using tri-block copolymers of Pluronic F108, P123 and F127 as structure-directing agents (SDAs) and temperature-controlled calcination (450–650 °C) has been carried out by direct templating-assisted hydrothermal approach. The structural/surface morphology and topographical properties of the photocatalyst are characterized using FE-SEM-EDAX, HR-TEM-SAED, p-XRD, VB-XPS, PLS, TG/DTA, UV-Vis-DRS, BET/BJH and zeta potential analysis. The undoped heterophase mesoporous TiO_2_ monoliths with in-built lattice/surface defects exhibit visible light photocatalytic properties, successfully dissipating Reactive Brown 10 (RB-10) dye. The influence of physicochemical parameters, such as SDAs, temperature, pH, dye concentration, catalyst dosage, photosensitizers and light intensities, are optimized for maximum photocatalytic performance at a shorter timespan. The F127-assisted mesoporous TiO_2_ monolith (550 °C) exhibits superior degradation kinetics (15 min) for RB-10 dye solution (20 ppm) at pH 2.0–3.0 using a photocatalyst dosage of 50 mg and 2 mM of KBrO_3_, irradiated with 150 W/cm^2^ tungsten lamp. The photocatalysts are fabricated without complicated chemical modifications and display topmost efficiency in quickly decontaminating persistent pollutants. The photoproducts from RB-10 photocatalytic degradation are investigated using HR-MS analysis. The photocatalyst can be reused efficiently for six cycles, even under extreme conditions.

**Supplementary Information:**

The online version contains supplementary material available at 10.1038/s41598-024-79285-3.

## Introduction

Ever since the report of TiO_2_ as a catalyst by Fujishima and Honda in the 1970s, the advanced oxidation processes using undoped TiO_2_ nanoparticles have been restricted to UV light and necessitate microfiltration to tackle issues of nanotoxicity. The discovery of mesoporous inorganic silica materials, such as MCM-41 and SBA-15, in the 1990s led to a new class of porous materials with unique structural properties. Mesoporous inorganic materials have been associated with high surface area and long-range porosity, which are imperative for adsorption, separation, catalysis, electrochemical, sensors, and energy conversions^1–3^. Despite the positives of TiO_2_, several chemical methods have been applied to enhance its visible light photocatalytic properties by tuning its morphology, shape, crystallinity and metal/non-metal doping by chemical/physical modifications^4–7^. Recently, efforts on synthesizing defect-based TiO_2_ are gaining favor over doped TiO_2_ for photovoltaics, photocatalysis, and electrochemical applications due to its narrow band gap (≤ 3 eV), allowing visible light harvesting without complicated modifications^8–11^. Introducing lattice defects in TiO_2_ exhibits relatively high electrical conductivity, enhancing its appeal for various applications.

In recent years, titania-based mesoporous materials have gained significant attention due to their better surface area, pore size, and structural topography than nanoparticles of TiO_2_^12–14^. Unlike Degussa P25, commercially available TiO_2_ nanopowders with a mixed-phase (anatase-rutile) optimized for photocatalytic applications. However, these nanoparticles are frequently associated with recovery and toxicity-related issues. Monolithic materials are highly effective alternatives to powdered catalysts due to their continuous, rock-like structure and parallel channels, facilitating superior mass transfer. The growing interest in mesoporous TiO_2_ monoliths for decontamination and energy harvesting applications suggests their utility as new-generation heterogeneous catalysts that are simple, inexpensive, and eco-friendly, with distinct electronic and optical properties^15,16^. In addition, researchers have made several advances in immobilizing TiO_2_ photocatalysts on different supporting matrices, such as foams, zeolite and resins prepared either through template-based polymers, tri-block copolymers, and surfactants or through template-free methods^17–19^. They offer improved thermal and mechanical stability, ease of recovery, reduced pressure drops, and enhanced catalytic activity, making them particularly advantageous for various catalytic processes^20–22^.

Novel porous TiO_2_ monoliths with tailored morphology, porosities, and architectural designs, particularly those exhibiting hierarchical porosity and mesopores in combination with macropores, are highly desirable for photocatalytic applications due to their unique structural advantages^1,23^. The structural consistency and porosity features of TiO_2_ monoliths make them effectively bind to the contaminants, boosting photocatalytic reactions^14,24,25^. Mesoporous monolithic TiO_2_ demonstrated superior physical properties for enhanced photocatalytic performance. The template-based hydrothermal synthesis offered a simple, cost-efficient, and eco-sustainable approach, making it particularly advantageous for large-scale industrial applications^26–28^. Hence, using a structurally engineered mesoporous TiO_2_ monolithic framework has significantly improved remediation and photo-/electro-chemical water splitting approaches^29^. Recent strategies have focused on utilizing a dopant-free TiO_2_ monolith as a visible light-responsive catalyst that can overcome the preexisting challenges in heterogeneous photocatalysis and present a promising alternative for improved process efficiency^30,31^.

In this work, we report on the temperature-controlled hydrothermal synthesis of mesoporous TiO_2_ monoliths using a direct templating liquid crystal phase micro-emulsion technique, using tri-block copolymers such as Pluronic F108, P123 and P127 as SDAs, in tuning the structural and surface morphological features. Reports on synthesizing TiO_2_ monolith by varying tri-block copolymers are available; however, we report a unique attempt at the micro-emulsion-mediated synthesis of visible light-responsive undoped mesoporous TiO_2_ monoliths by hydrothermal process and a detailed analysis of the photocatalytic efficiency of the resultant TiO_2_ monoliths. In this work, the effects of SDAs on the structural morphology, pore size, the surface area of the TiO_2_ monoliths are comprehensively characterized, and the potential application of these monoliths towards the photocatalytic degradation of RB-10, a widely employed azo-based fabric dye has been investigated. The synergistic combination of rutile and anatase of TiO_2_ has been widely reported to improve photocatalytic performance, primarily due to creating a staggered band gap at the phase junction that promotes charge separation, thereby enhancing photocatalytic efficiency. Although the precise direction of charge migration across the phase junction remains under investigation, it is generally accepted that electrons migrate from the rutile to the anatase phase while holes move from anatase to rutile. Hence, we have chosen TiO_2_ with the advantages features of heterophase composition and unique properties of monolith structure^30,31^. The synthesized TiO_2_ monoliths exhibited efficient photo-oxidative properties with variation in their performance under visible light irradiation. F127-based mesoporous TiO_2_ monoliths with a highly porous architecture proffered large surface area and enhanced adsorption properties.

In this work, an in-depth study on the degradation pattern of RB-10 has been carried out to establish a reliable decontamination approach for textile dye effluents. Hence, we performed a systematic study on the influence of various experimental parameters by varying the solution pH, dye concentration, photocatalyst dose, kinetics, sensitizer/oxidizers, and light intensity to ascertain the optimal conditions for enhancing the photocatalytic efficacy using the best visible light responsive mesoporous TiO_2_ monolithic photocatalyst from the monoliths synthesized by varying the SDAs and calcination temperature.

## Results

### Analytical characterization of mesoporous monolithic photocatalyst

#### p-XRD analysis

**Fig. 1:**
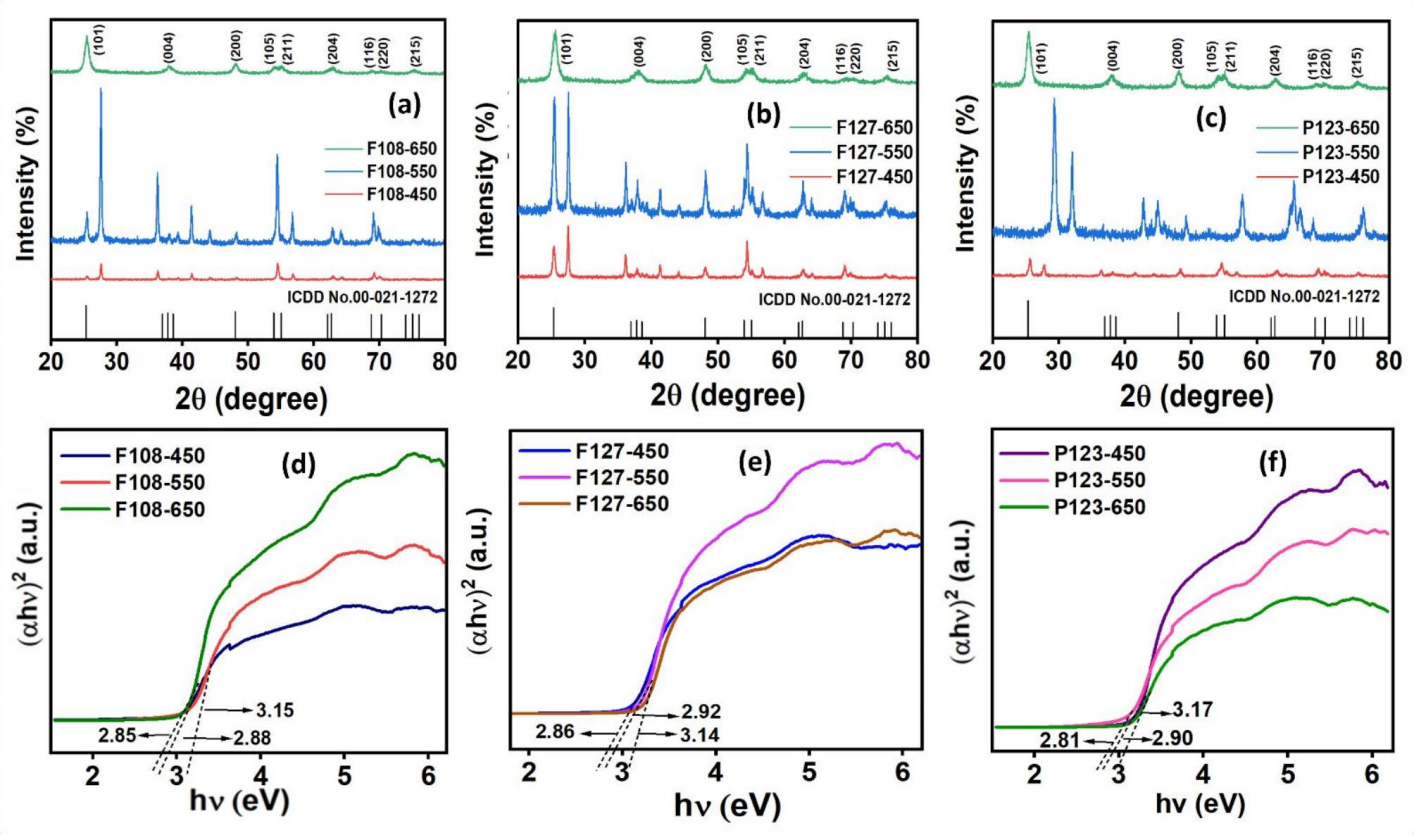
**a–c** p-XRD pattern and **b** Tauc plot for F108, F127 and P123 SDAs based mesoporous TiO_2_ monoliths calcinated at 450 °C, 550 °C and 650 °C.

The p-XRD patterns of TiO_2_ monolith synthesized by varying the triblock copolymers (F108, P123 & F127) and calcination temperatures (450 °C, 550 °C & 650 °C) have been depicted in Fig. 1a–c. The p-XRD patterns of F108-based TiO_2_ monolith synthesized at calcination temperatures of 650 °C and 550 °C revealed the presence of a crystalline mixed phase of anatase and rutile peaks (2θ) at 25.2º and 28.1º corresponding to the h k l reflections of (1 0 1) and (1 1 0) planes, with a prominent presence of rutile phase, as depicted in Fig. 1a. However, the F108-450 TiO_2_ monolith sample revealed the presence of a pure anatase phase and the absence of a rutile phase, indicating the significance of calcination temperature for TiO_2_ monolith synthesis. Figure 1b demonstrated the phase analysis of F127-based TiO_2_ monoliths, where the F127-650 and F127-550 samples showed mixed ratios of anatase and rutile phase, with the rutile phase being prominent at higher temperatures (≥ 650 °C). The F127-550 samples showed an equal intensity of rutile and an anatase phase, and the F127-650 sample revealed a low anatase phase and intense rutile phase peak. The p-XRD pattern (Fig. 1c) of P123-based TiO_2_ monoliths showed anatase predominance over the rutile phase even at calcined temperatures of 550 °C and 650 °C. However, the p-XRD data revealed a pure anatase peak for P123-450 TiO_2_ samples without any phase transitions to the rutile phase. The phenomenon led to an inference that the heterophase formation (anatase and rutile) for F108, F127 and P123-based TiO_2_ monoliths occurs at 550 °C/650°C, whereas the pure anatase phase exists at 450 °C, irrespective of the nature of SDA used for TiO_2_ monolith synthesis. It is known that the anatase phase exhibits activity only under UV light, while the rutile can be activated in visible light to its ratio to the anatase phase. The coexistence of anatase and rutile phases in TiO_2_ monolith at 550 °C proved to be a pivotal factor in establishing the synergetic effect with intermediate energy levels derived from the lattice/surface defects and the relative position of conduction band edges in the two phases in contact (interface) that favor visible light photocatalysis^32,33^. The photocatalytic performance can be improved as the TiO_2_ heterophase can competently carry charge carriers across phases. The TiO_2_ homophase with solely anatase resulted in a high recombination rate, low adsorption and low thermal ability due to the cumulative effect of crystallographic and microstructural factors. On the contrary, samples with dual anatase-rutile phases have more discernible crystallography and nanostructure between anatase and rutile, which diminishes the recombination rate of charge carriers. The phase transformations and the presence of well-contacted heterophase interfaces can be achieved through precise control of the synthesis conditions and temperature-controlled calcination.

In the subsequent photocatalysis studies, the samples that exhibited a mixed proportion of anatase and rutile phases, particularly at 550 °C, exhibited superior visible light absorption properties. The possible reason was inferred from the Debye-Scherer formula, where the average crystallite sizes of F127-550, F108-550 and P123-550 of TiO_2_ monolith were about 36.0 nm, smaller than TiO_2_ monoliths synthesized at 650℃ (38.6 nm). The p-XRD data revealed that the crystallite sizes and phases of the TiO_2_ monoliths varied as a function of calcination temperature. Larger crystallite sizes at higher temperatures were attributed to the faster growth of crystallites. Hence, in the case of F127-550 TiO_2_, the lower crystallite size led to the significant formation of heterophase with oxygen vacancies/lattice defects that act as intermediate energy levels for photocatalysis by facilitating efficient charge transfer/separation and accelerated decontamination of the pollutant molecules through photogenerated reactive oxygen radical species. The voluminous generation of radical intermediates with reduced crystallite size has been ascribed to the quantum confinement effect that enhanced the visible light photocatalytic performance^34,35^. The visible light photocatalysis was absent for TiO_2_ monoliths prepared at 450 °C despite their smaller crystallite size (~ 34.1 nm) due to the UV light active anatase phase. The F127-550 TiO_2_ monolith was inferred to exhibit excellent visible light photocatalytic properties due to crystallite sizes and heterophase (anatase-rutile) variations that offered intermediate energy states for efficient charge carrier entrapment. Hence, the particle size and crystallinity controlled the migration rate of the charge carriers. Further details on the p-XRD data obtained for the various SDAs-based TiO_2_ monolith at 550 °C have been included in Table S1 (Electronic Supplementary Material).

#### UV-Vis-DRS and PLS analysis

The UV-Visible diffuse reflectance spectral (UV-Vis-DRS) measurements to study the light absorption properties of the TiO_2_ monoliths from different SDAs (F108, F127 and P123) and calcination temperatures (450 °C, 550 °C and 650 °C) have been depicted in Fig. 1d–f. The variation in the light absorption properties as a function of wavelength proved crucial in accessing the photocatalytic behavior of the synthesized TiO_2_ monoliths. The energy band gap measurement has been an influential parameter, where a lower energy band gap enhances the visible light photocatalytic activity for effluent dissipation. For the synthesized mesoporous TiO_2_ monolithic photocatalysts, the optical energy band gap values have been calculated using Tauc’s plot (extrapolated from the Kubelka-Munk function), as shown in Eq. (1).

1 $$\alpha {\text{h}}\nu \,=\,{\text{A }}{({\text{h}}\nu - {{\text{E}}_{\text{g}}})^{\text{n}}}$$

Here, ‘α’ denotes the attenuation constant, ‘A’ is a constant, ‘h’ is Planck’s constant, ‘n’ is 0.5 for indirect transitions, ‘ν’ represents the frequency of light and ‘E_g_’ represents the energy band gap. The UV-Vis-DRS plot revealed a narrowing energy band gap with increasing calcination temperature for the TiO_2_ monoliths prepared from different SDAs. The titania monoliths synthesized at 450 °C exhibited an energy band gap range of 3.14–3.15 eV, primarily associated with the light absorption in the UV region. However, the P123-550, F127-550 & F108-550, and P123-650, F127-650 & F108-650 samples show an energy bandgap range of 2.81–2.92 with better visible light absorption. A red shift in the energy band gap with increasing calcination temperature can be attributed to the quantum confinement effect, causing an increased formation rate of electron-hole pairs on the photocatalyst surface, with improved photocatalytic activity^36,37^. However, considering the crystallite size and lattice strain parameters as a function of temperature, better results were achieved using F127-550-based TiO_2_ monoliths that were in good coherence with the p-XRD data. Hence, these resultant energy band gaps were associated with the nature of phase compositions, crystalline sizes, lattice strains and particle sizes, which led to the F127-550 TiO_2_ monolith exhibiting better visible light photocatalysis. The optical energy band gap values for the TiO_2_ monoliths synthesized using various SDAs and calcination temperatures have been tabulated in Table S2 (Electronic Supplementary Material).

The photocatalytic behavior of the catalyst, its degradation of the target species, and the proportion of photoactive sites in the catalyst can be studied using photoluminescence spectra (PLS). The PLS analysis for TiO_2_ monoliths derived from different SDAs and calcination temperatures was carried out at an excitation wavelength of 300 nm to analyze their emission peak intensities to ascertain the extent of electron-hole pair recombination effect on the photocatalysis process (Fig. S1, Electronic Supplementary Material). The PLS plot revealed that greater peak intensity directly reflected the significant recombination effect, thus weakening the photocatalytic activity^38^. In the case of the synthesized TiO_2_ monoliths, the structural defects, such as oxygen interstitials and vacancies, led to various radiative transitions between photoexcited electrons at the conduction band/trapping levels and the photogenerated holes at the valence band/intermediate trapping levels^39^. The samples’ temperature conditioning boosts the oxygen defects, further suppressing the PL intensity and improving the charge carrier separation efficiency of the photoexcited electrons and holes. The connexion crystalline heterophase of TiO_2_ generates an internal electric field that favors the separation of photoinduced e^−^/h^+^ pairs. Compared to all samples, the F127-550 TiO_2_ monolith showed the lowest PLS peak intensity, representing the excess presence of non-recombined numbers of electrons and holes that, in turn, facilitated the creation of hydroxyl and superoxide radical intermediates for the effective photocatalytic dye degradation. However, PL intensities decreased with increasing temperatures from 450 to 650 °C.

The high emission peak intensity observed at the near band edge around 472 nm corresponds to the electron-hole pair recombination factor. The broad emission peak at 472 nm was observed due to vacuity in the interstitial sites of the TiO_2_ monolith. Due to the dissimilarities in the band gap energy of different titania monoliths, a slight difference in the emission peak pattern was observed. However, the TiO_2_ monolith derived from the F127-550 combination showed the lowest PLS intensity compared to other SDA-based TiO_2_ monoliths, making it a suitable candidate for visible light photocatalysis. The visible light photocatalytic properties of the mixed phase F127-550 TiO_2_ monoliths can be achieved with an interconnected heterophase interface for efficient interfacial charge transfer and fine-tuning of surface area, particle size and oxygen vacancies.

#### BET/BJH plots and TG analysis

**Fig. 2:**
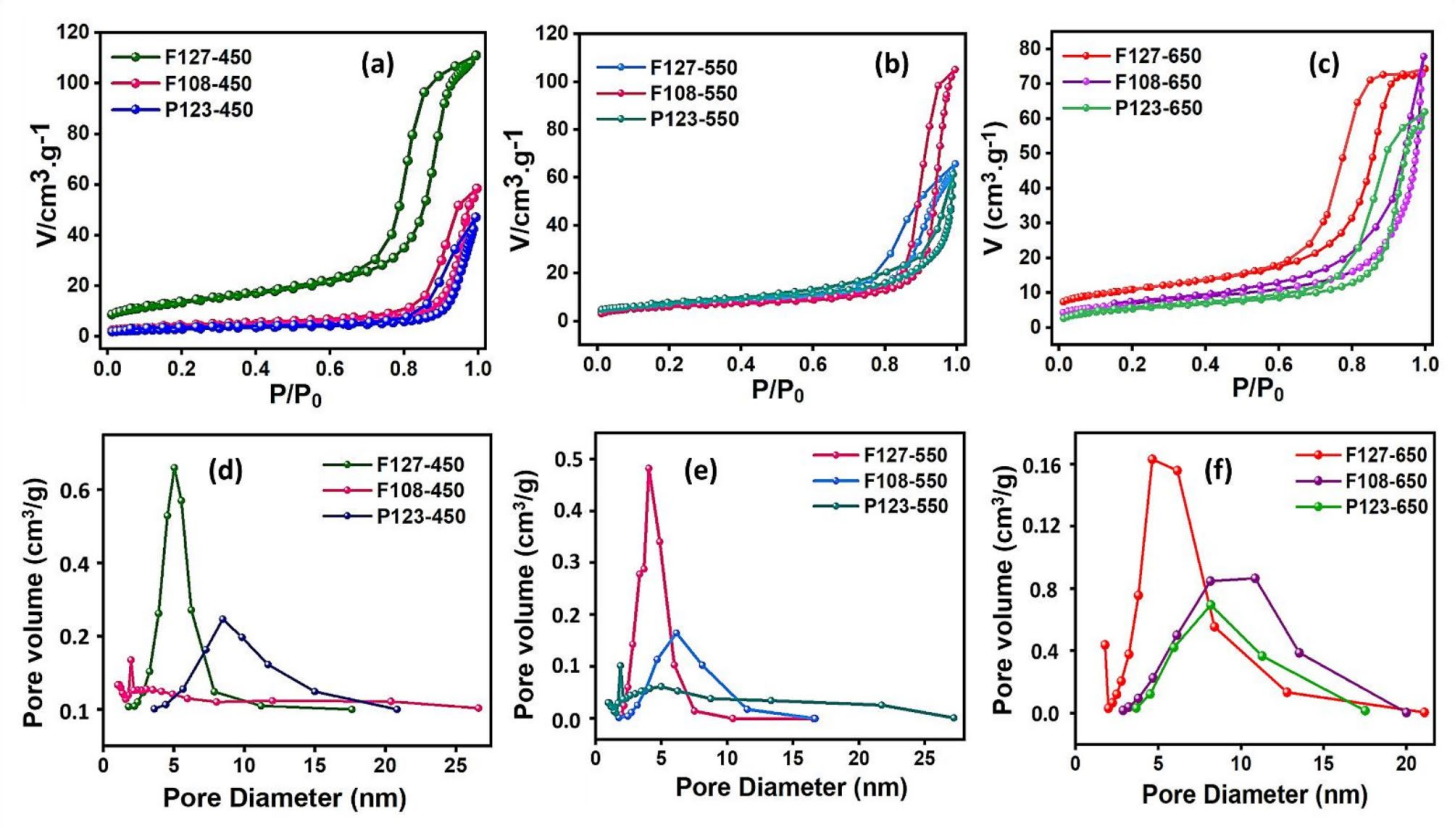
**a–c** N_2_ adsorption-desorption isotherm pattern and **d–f** BJH Pore size distribution of F108, F127 & P123 SDA-based TiO_2_ monoliths at 450 °C, 550 °C & 650 °C respectively.

The surface area measurements using N_2_ adsorption-desorption isotherm analysis (BET) and the pore size distribution plots (BJH) were performed for TiO_2_ monoliths prepared using different SDAs and varying calcination temperatures, as depicted in Fig. 2a–c and d–f, respectively. The isotherm pattern indicated that the monolith samples exhibited an H_1_ hysteresis loop, with a cylindrical porous network revealing a type V isotherm pattern, which indicates a continuous network mesoporous structural framework^40^. The BET surface area and pore dimensions have been tabulated in Table S3 (Electronic Supplementary Material). From the isotherm pattern obtained, the F127 SDA-based TiO_2_ monolithic samples exhibit greater surface area than F108 and P123 SDAs-based TiO_2_ monoliths. Besides, due to decreasing crystallite sizes at higher temperatures, high surface area and better porosity features were noticed at 450 °C and 550 ºC compared to 650 ºC. The declining surface area and mesoporosity with increasing calcination temperature, irrespective of the nature of the SDA deployed, has been attributed to the increasing particle size associated with the severe collapse of the initial porous network of the monolithic structure at high temperatures, as seen in the BJH pore size distribution data. As indicated in the p-XRD data, an increase in calcination temperature decreases the average pore volume and crystallite size^41^. Hence, surface free energy dominates below a specific particle size, energetically stabilizing the anatase phase at higher temperatures. Based on the BET/BJH plot, it has been concluded that the TiO_2_ monolith derived from F127-550 exhibited better surface and pore properties. The slight increase in the pore volume of the TiO_2_ monolith characterized by a type V isotherm pattern created a favorable condition for the enhanced assimilation of the dye pollutants followed by the light-induced dye dissipation process.

In this line, the thermogravimetric analysis (TGA) data for the TiO_2_ monoliths synthesized using F127 SDA, calcined at different temperatures has been depicted in Fig. S2 (Electronic Supplementary Material). The data indicate that F127 SDA-based TiO_2_ monoliths have better water retention capacity across different calcination temperatures. The hydrophilic character led to excellent water percolation through the mesoporous channels, thus leading to significant interaction of dye molecules with the photoactive sites in the monolith framework, paving the way for possible faster dye degradation through the rapid and voluminous generation of reactive oxygen species radicals, signify for improved photocatalytic performance.

#### FE-SEM- EDAX and HR-TEM-SAED analysis

**Fig. 3:**
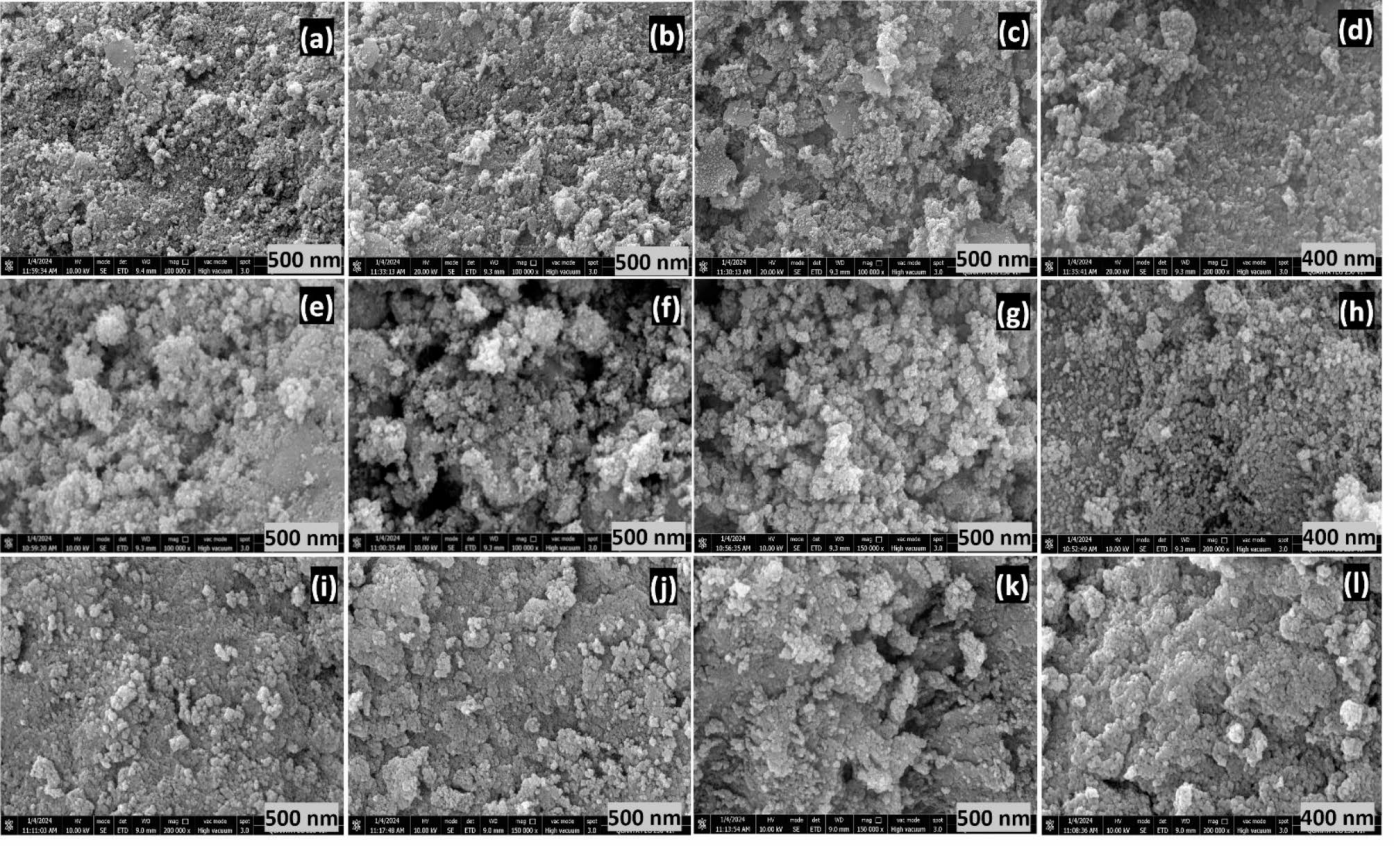
FE-SEM images of **a**–**d** F108, **e**–**h** F127 and **i**–**l** P123 SDAs-based TiO_2_ monolith at 550 °C.

The FE -SEM-EDAX analysis of the monoliths with different SDAs (F108, F127 & P123) at 550 °C has been illustrated in Fig. 3a–l. An increase in crystallite and pore sizes of the monolithic materials has been noticed (in FE-SEM images) as a function of calcination temperatures, suggesting enrichment in the crystallite growth of titania monoliths, as supported by the p-XRD analysis. The larger size of the mesoporous may be due to the aggregation of primary particles due to the nucleation of the TiO_2_ framework from the hydrolysis of titanium isopropoxide that led to the formation of mesoporous titania monoliths by thermal crystallization. Sintering the inorganic precursors while forming a continuous framework has increased the crystallite size. FE-SEM images also confirmed the presence of a mesostructured framework by indicating the pore shrinkage during calcination. The images also show the collapse of pore channels where TiO_2_ monoliths were identified as spherical and some particles agglomerated together to clusters at 650ºC, which shows that samples calcined beyond 550 °C possess poor surface morphology and smaller pore volume^42^.

**Fig. 4:**
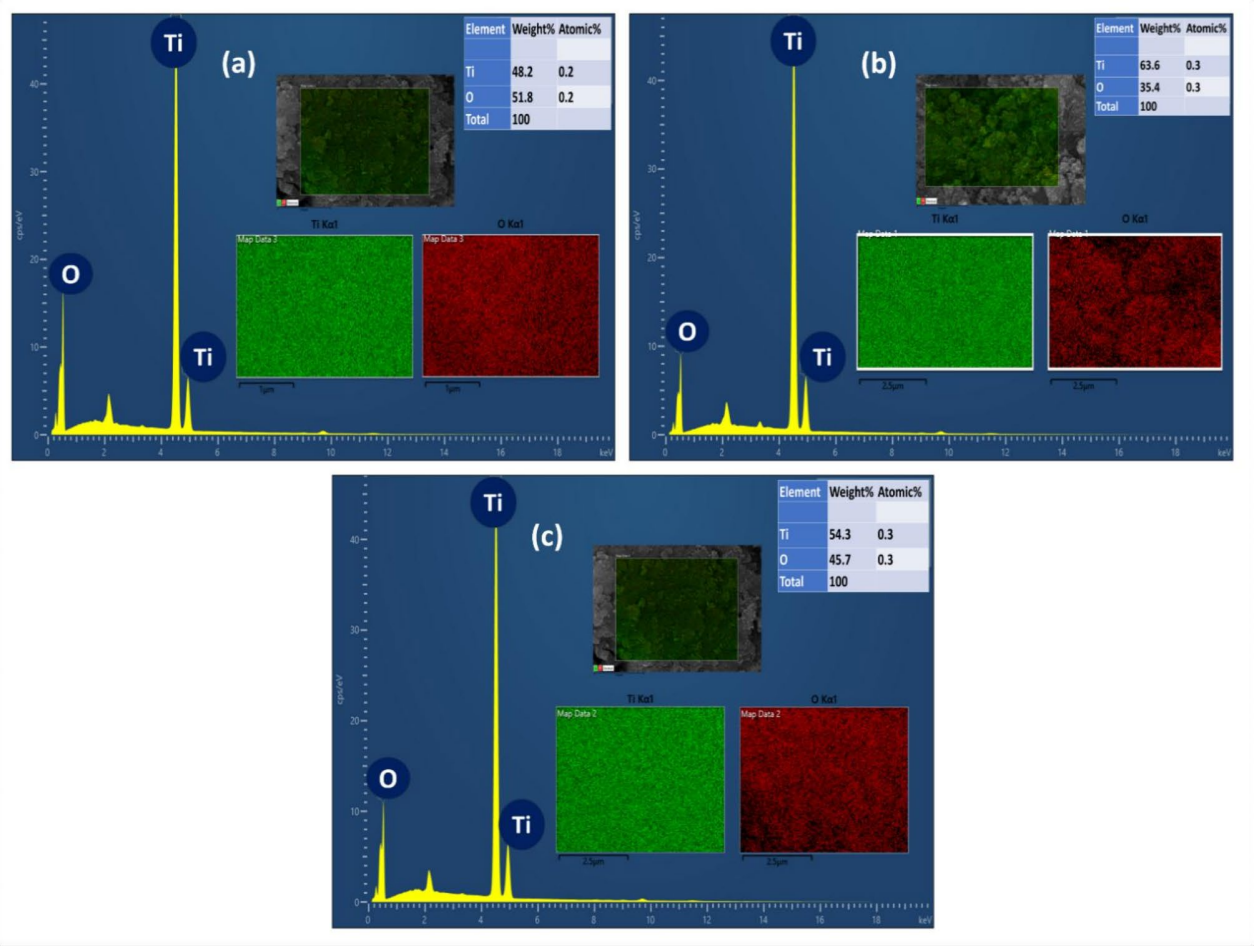
**a**–**c** EDAX and elemental mapping (*inlet images*) for various SDAs-based TiO_2_ monoliths at 550 °C.

Figure 4a–c depicted the EDAX pattern confirming the elemental presence of Ti and O, with their relative mass/atomic ratios of TiO_2_ monolith derived using varying SDAs of F108, F127 and P123, respectively, at a calcination temperature at 550 °C. The elemental mapping images confirmed the long-range homogeneous network of the porous TiO_2_ monolith. EDX and elemental mapping analysis have confirmed the absence of carbon in the F127-550 Ti monolith to ascertain the involvement of heterophase (anatase-rutile) of TiO_2_ towards visible light photocatalytic activity.

**Fig. 5:**
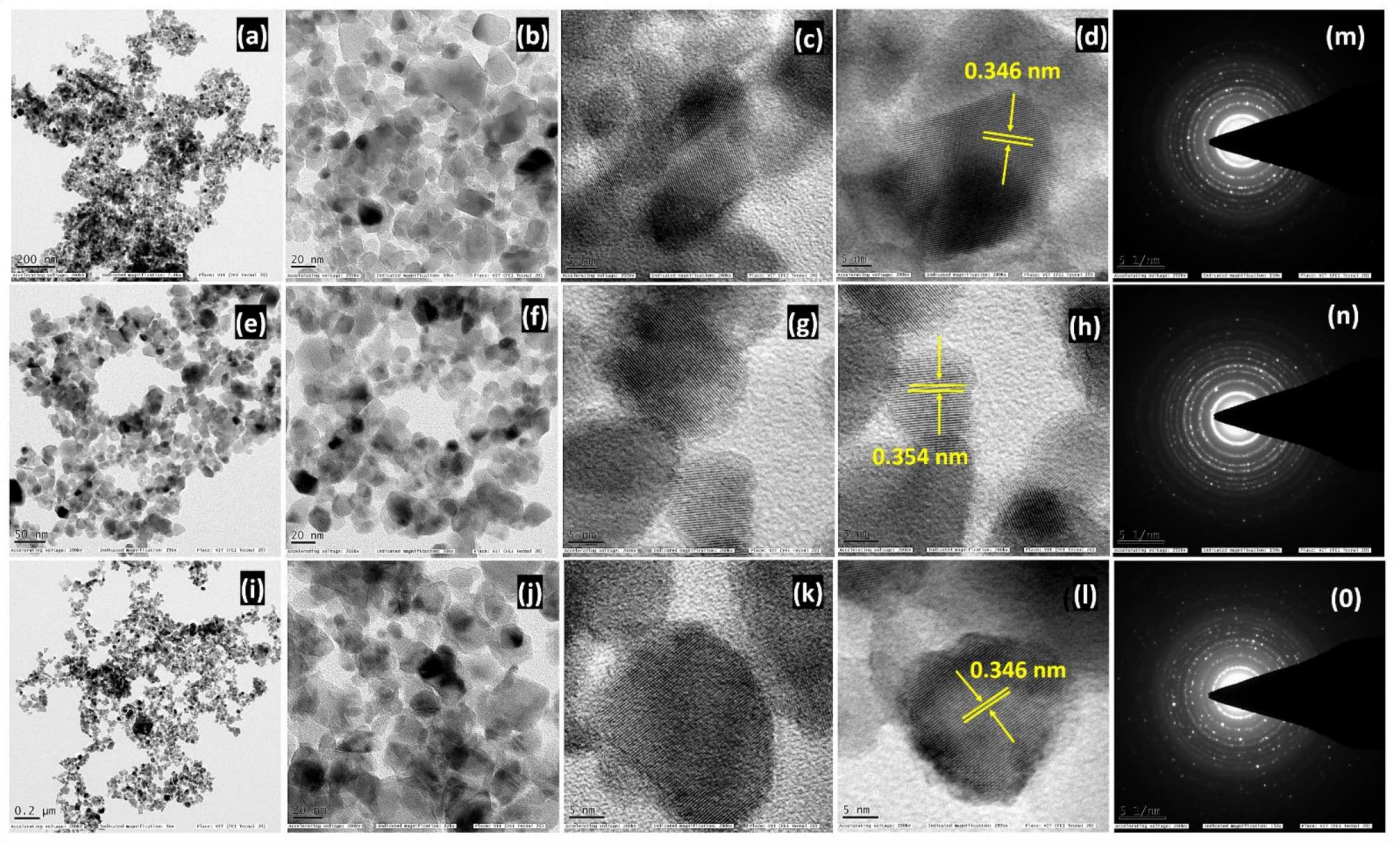
TEM, HR-TEM of **a–d** F108, **e–h** F127 and **i–l** P123 SDA-based TiO_2_ monoliths at 550 °C. **m–o** SAED patterns of F108, F127 and P123 SDAs based mesoporous TiO_2_ monoliths at 550 °C.

The structural and morphological traits of F108-550, F127-550 and P123-550-based TiO_2_ monoliths were analyzed by TEM, as shown in Fig. 5a–c, e–g and i–k. The TEM images for the monoliths confirmed the interconnected mesoporous channels of a continuous network with high surface area and pore dimensions. The HR-TEM images show a set of lattice fringes with an interplane spacing of 0.321 nm has been observed for the F108-550 TiO_2_ monolith (Fig. 5d) that corresponds to its predominant rutile phase. In the case of the F127-550 TiO_2_ monolith (Fig. 5h), the HR-TEM images reveal lattice fringes with spacings of 0.354 and 0.327 nm assigned to (0 0 1) and (1 0 1) facets of TiO_2_ anatase and rutile phases denoted as A (0.354) and R (0.327), respectively, thus confirming the presence of a mixed phase. For TiO_2_ monolith derived from P123-550 (Fig. 5l), the d value of 0.346 nm corresponds to the (1 0 1) plane to represent the predominate existence of TiO_2_ monolith in its anatase phase, which matches with the p-XRD results. The SAED Fig. 5m–o pattern for F108-550, F127-550 and P123-550 TiO_2_ monoliths showed bright spots, indicating the crystalline nature of the TiO_2_ monolith, which corroborated with the p-XRD data.

#### XPS analysis

**Fig. 6:**
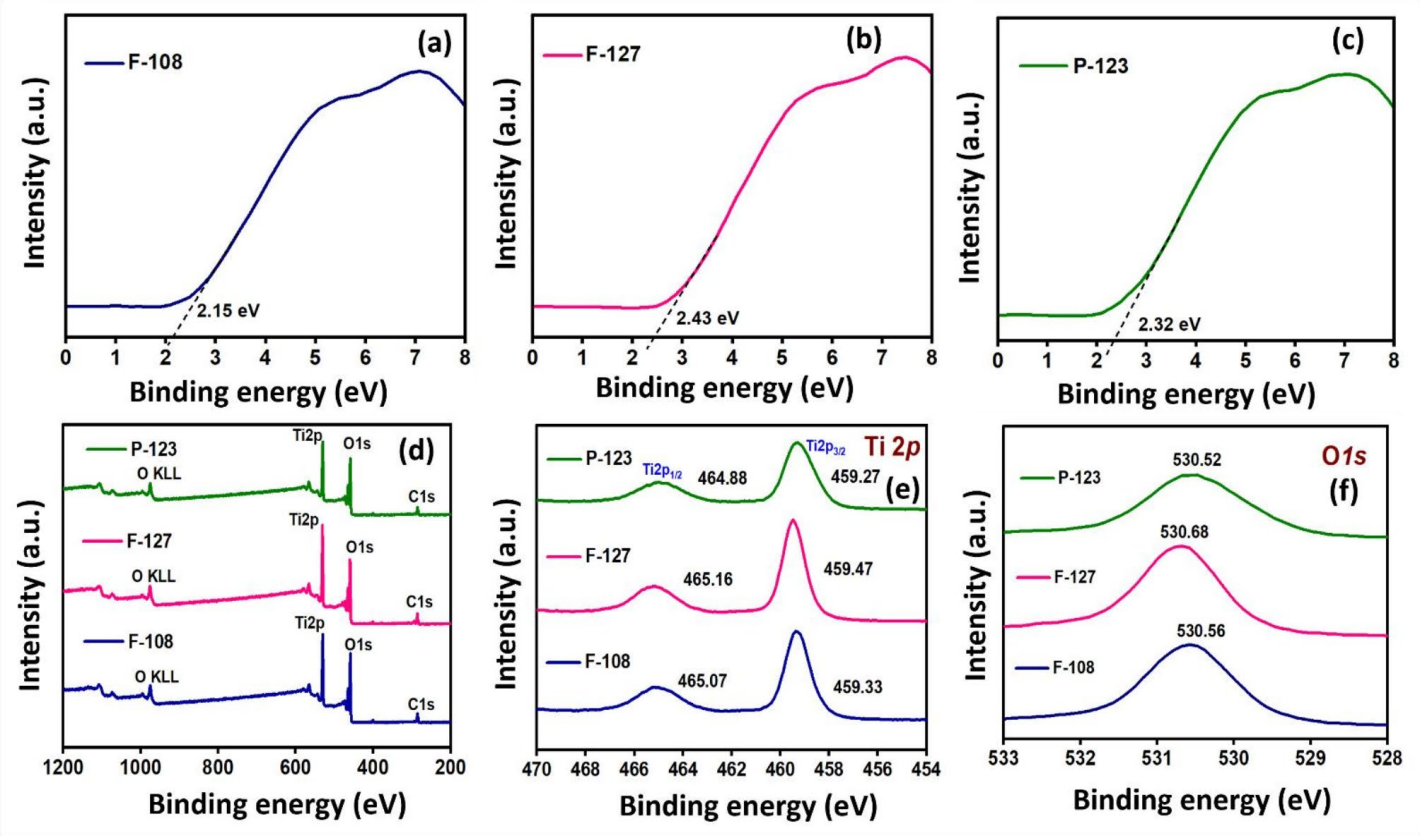
**a–c** VB-XPS spectra, **d** Full-scan XPS and **e–f** High-resolution deconvoluted XPS pattern for Ti*2p* and O*1s* orbital states of F108, F127 and P123 SDAs based mesopore TiO_2_ monoliths calcinated at 550 °C.

The effect of different surfactants on TiO_2_ monolith at 550℃ toward the surface element compositions and states over the prepared catalysts is examined by XPS characterization. The energy band structure for light-driven photocatalysis was determined using VB-XPS measurements for F108-550, F127-550 and P123-550 monoliths, as illustrated in Fig. 6a–c. The complete survey spectra Fig. 6d of the F108-550, F127-550 and P123-550 monolith revealed the characteristic elemental peaks of Ti and O that were consistent with the EDS mapping analysis. The high-resolution spectrum for the Ti*2p* orbital state in the binding energy range of 454–470 eV presents two peaks for F108-550, F127- 550 and P123-550 monolith, as shown in Fig. 6e. The two deconvoluted peaks at 459.33 and 465.07 eV for F108-550, 459.47 and 465.16 for F127-550 and 459.27, 464.88 for P123-550 are assigned to the Ti*2p*_*3/2*_ and Ti*2p*_*3/2*_ orbital energy states of TiO_2_. The shift in the Ti*2p* orbital state peak suggests the formation of lattice defects of Ti^3+^, responsible for energy bandgap narrowing. The photocatalytic behavior of TiO_2_ was increased by the presence of Ti^3+^ defects combined with the lattice distortion. The high-resolution O1s spectrum is depicted in Fig. 6f, revealing prominent peaks at 530.56 eV, 530.68 eV, and 530.52 eV of F108-550, F127-550, and P123-550 monoliths, respectively.

The high-resolution deconvoluted XPS data for the Ti*2p* orbital state present three peaks in the binding energy range of 457–468 eV for P123, F127 and F108 SDA-based TiO_2_ monoliths at 550 °C, as shown in Fig. S3a (Electronic Supplementary Material). The two deconvoluted components located at 459.3 and 464.9 eV for F108-550, 459.5 and 465.1 eV for F127-550 and 459.1, 464.9 for P123-550 were assigned to Ti*2p*_*3/2*_ and Ti*2p*_*1/2*_ orbital states indicating the existence of + 4 oxidation state of Ti. The peaks at the binding energy region of 459.3, 459.6 and 459.4 eV correspond to the Ti*2p*_*1/2*_ in + 3 oxidation state of Ti_2_O_3_ for F108-550, F127-550, and P123-550, respectively. The shift in the Ti*2p* peak suggests the presence of lattice defect-induced Ti^3+^ that contributes to the bandgap narrowing. The photocatalytic behavior of TiO_2_ was enhanced by the presence of Ti^3+^ defects combined with the lattice distortion. In this line, the high-resolution deconvoluted O*1s* spectra of TiO_2_ monoliths from F108, F127 and P123 SDAs at 550 °C as depicted in Fig. S3b (Electronic Supplementary Material). The deconvoluted high-resolution O*1s* spectra revealed binding energy peaks at 531.0, 531.7, and 531.1 eV corresponding to the lattice oxygen of P123-550, F127-550 and F108-550 TiO_2_ monoliths. Similarly, the binding energy peaks at 530.4, 530.7, and 530.5 eV correspond to the oxygen species of Ti_2_O_3,_ and the binding energy peaks at 529.9, 530.3 and 530.1 eV belong to the surface adsorbed hydroxyl oxygen of F108-550, F127-550, and P123-550 TiO_2_ monoliths. The geometrical imperfections caused by lattice/surface defects benefit visible-light absorption, facilitating potential visible-light responsive photoactive properties for the heterophase TiO_2_ monolith photocatalysts^43–45^, as inferred from the E_g_ and PLS data. These defects distort the TiO_2_ monolith geometry and influence the charge carrier’s transport/separation, thus playing a significant role in band gap narrowing for visible light photocatalysis, predominantly for the F127-550 TiO_2_ monolith.

### Impact of operational parameters on photocatalysis

**Fig. 7:**
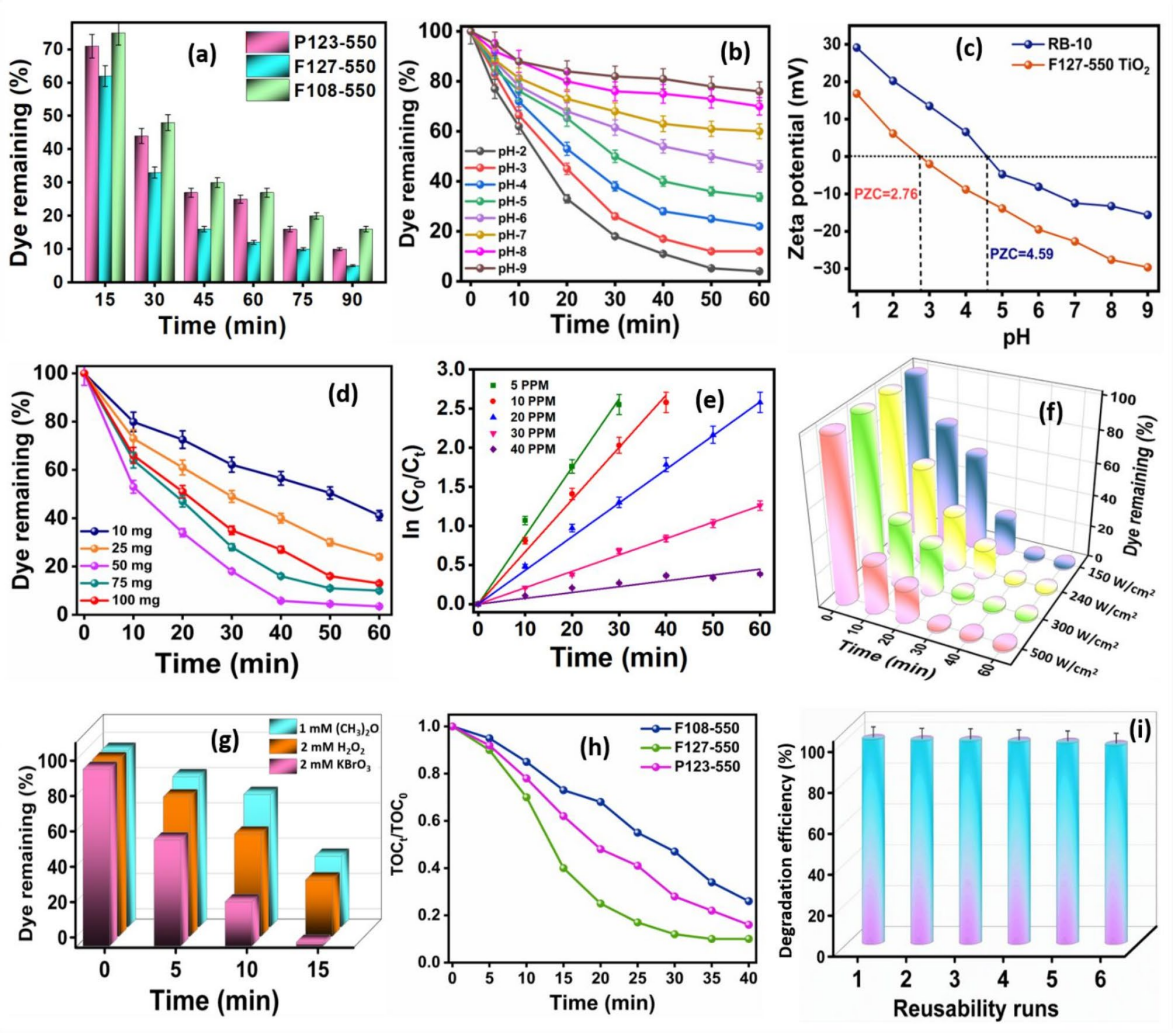
**a** Influence of SDAs’ on TiO_2_ monolith synthesis at 550 °C for visible light photocatalysis of RB-10. Effect of **b** Solution pH, **c** Zeta potential, **d** Photocatalyst dosage, **e** Dye concentration, **f** Visible light intensities, and **g** Sensitizers/Oxidizers using F127-550 TiO_2_ monolith on the photocatalytic degradation of RB-10 dye. **h** TOC analysis of RB-10 dye mineralization using F127-550, P123-550 and F108-550 TiO_2_ monoliths. **i** Reusability study using F127-550 TiO_2_ monolith photocatalyst for RB-10 degradation.

The pH-dependent photocatalytic performance of the undoped mesoporous TiO_2_ monoliths (P123-550, F127-550 and F108-550) were assessed through RB-10 dye degradation under visible light irradiation, as shown in Fig. 7a. From the plot, the F127-550 TiO_2_ monolith showed ≥ 92% RB-10 dye degradation in 1.5 h compared to P123-550 and F108-550 TiO_2_ monoliths. The visible light photocatalysis behavior was attributed to energy bandgap narrowing that accelerates the electron-hole pair generation, leading to charge carrier separations in the lattice/surface defects, eventually enhancing the degradation process through the reactive oxygen species. Hence, the F127-550 TiO_2_ monolith was used for all subsequent photocatalysis experiments.

The effect of solution pH on photocatalytic degradation utilizing RB-10 dye solution with F127-550 TiO_2_ monolith was investigated to identify the suitable pH condition to extract maximum dye degradation. Figure 7b demonstrates the degradation profile for 20 ppm of RB-10 dye solution under visible light irradiation after equilibration with F127-550 TiO_2_ monolith in the pH range of 2.0–9.0. Besides, the zeta potential was measured at various pH to elucidate the isoelectric point of the photocatalyst, as depicted in Fig. 7c. For RB-10 and F127-550 TiO_2_ monolith, an isoelectric point (point of zero charge) was observed at pH 2.76 and 4.59, respectively, which paved for maximum adsorption and subsequent dissipation of RB-10 dye molecules onto the F127-550 TiO_2_ photocatalyst in the pH range of 2.0–3.0. The maximum photocatalytic degradation in the pH range mentioned above was attributed to the surface charge of the photocatalyst and the ionic state of RB-10 molecules. At pH 2.0, the dye degradation is more prominent due to the positively charged F127-550 TiO_2_ monoliths that create a solid electrostatic force of interaction with the negatively charged sulfonic acid groups of the RB-10 molecules, promoting effective dye adsorption onto the photocatalyst surface, thereby facilitating faster visible light induced photocatalytic degradation. The slow dye degradation rate at increasing solution pH was attributed to the zwitterionic and anionic state of existence of RB-10 dye molecules, which explains its greater tendency to retain in an aqueous medium rather than interacting with the negatively charged TiO_2_ surface monolith. At pH ≥ 4.0, i.e., beyond the isoelectric point, the predominant existence of the negatively charged photocatalytic surface of the F127-550 TiO_2_ has prompted the electrostatic repulsion with the anionic RB-10 molecules, thereby decreasing their absorption onto the photocatalyst surface, for degradation.

Figure 7d illustrates the optimal quantity of photocatalyst required for efficiently degrading RB-10 dye solutions of varying concentrations. A series of studies with varying quantities of F127-550 TiO_2_ monolith ranging from 10 to 100 mg was equilibrated with 20 ppm of RB-10 dye solutions (100 mL; pH 2.0). The data indicate an increase in the degradation efficiency with increasing catalyst dosage, with a maximum response at 50 mg due to the greater availability of photoactive sites for voluminous adsorption and dissipation of dye molecules onto the photocatalyst surface area. However, beyond 50 mg, a slight decline in the dye degradation kinetics was observed due to the aggregation of monolith materials with excess dosage in the solution medium, decreasing the process efficiency through the scattering effect of incident photons, thus preventing successful photocatalysis. The results reveal the requirement for minimal quantities of photocatalyst for the faster and more efficient degradation/dissipation of pollutant molecules through enhanced adsorption. The optimal dose processes the voluminous generation of reactive oxygen radical intermediate through the efficient interaction of incident photons with the photoactive sites, encouraging faster dye degradation efficiency.

The influence of RB-10 concentration on the extent of photocatalytic degradation of F127-550 TiO_2_ monolith and the eventual kinetics factor have been depicted in Fig. 7e. Hence, dye concentrations in the 5–40 ppm range were equilibrated under optimum conditions, and the dye dissipation was analyzed as a function of time. The data representations reveal a pseudo-first-order kinetics, and dye degradation efficiency increases with decreasing pollutant concentrations. In other words, with the increasing presence of dye molecules in the aqueous solution, a significant percentage of incident photons interact with the dye molecules, leading to photoexcitation. Thus, the dye molecules (beyond a critical concentration) exhibit a shielding effect for the incident photons, thus preventing its interaction with the photocatalyst to produce charge carriers. Hence, an increase in the time factor for the dye degradation, particularly with higher dye concentrations (≥ 30 ppm), has been attributed to the path length hindrance to the incident photon penetrating through the aqueous solution. The light-obscuring factor caused by the increasing dye concentration shortens the photons’ trajectory to interact with the photocatalyst’s photoactive site, lowering the photocatalytic efficacy. In photocatalysis, the rate-determining step has been associated with the adsorption of the pollutant molecules onto the photocatalyst surface and eventual photocatalytic degradation. From the kinetics plot, it has been deduced that at lower dye concentrations, due to the greater availability of the photocatalyst surface, faster dye degradation kinetics has been observed within 1 h of visible light-induced photocatalysis. Hence, considering the real-time utility and practical convenience, a 20 ppm RB-10 dye solution (as potential effluent concentration) has been adopted for subsequent photocatalytic studies using F127-550 TiO_2_ monolithic photocatalyst.

The effect of visible light intensity irradiated onto the photocatalyst for the degradation of RB-10 dye has been evaluated under different light intensities (150, 240, 300 and 500 W/cm^2^ tungsten filament lamp), as illustrated in Fig. 7f. Under optimized conditions, at 150 and 240 W/cm^2^ light intensity, ≥ 98.5% degradation was observed within 1 h. At lower light intensities, the photocatalytic degradation kinetics exhibits a linear response due to the competition between electron-hole pair formation and its recombination effect. In the case of higher light intensities, i.e., 300 & 500 W/cm^2^, faster dye degradation kinetics of > 98% was observed in 40 min of irradiation. At higher light intensities, the voluminous generation/separation of electron-hole pairs nullifies the recombination effect, thereby increasing the formation of reactive oxygen radicals and holes that serve as active species for the degradation of the RB-10 dye molecules. However, higher light intensity (300 & 500 W/cm^2^) results in significant wastage of energy apart from thermal pollution that can be avoided by adopting lower light intensities, i.e., 150 W/cm^2^ visible light that can simulate a natural sunlight condition for pollutant dissipation.

The impact of photosensitizers/oxidizers on the RB-10 dye degradation using F127-550 TiO_2_ monolith under visible light illumination has been indicated in Fig. 7g. From the graph among all the oxidizers, under optimized conditions, potassium bromate induces a faster degradation rate RB-10 (≥ 98.7%; 20 ppm; pH 2.0; 50 mg) within 15 min of photocatalytic irradiation, compared to using (2 mM) acetone and hydrogen peroxide as sensitizers. From the graph, ≥ 95% of RB-10 dye degradation using (1 mM) acetone and (2 mM) H_2_O_2_ could be achieved within 40 min of irradiation under identical conditions. The effect of KBrO_3_ concentration on the photodegradation process revealed that 2 mM of KBrO_3_ inclusion into the reaction medium was sufficient to achieve ≥ 98.7% RB-10 degradation in ≥ 15 min. The development of highly active radicals and their associated electron capture capability led to a faster photocatalytic deterioration of the dye molecules in the presence of photosensitizers. The degradation efficiency decreases beyond the optimized concentrations (2 mM H_2_O_2_, 1 mM acetone, and 2 mM KBrO_3_) of the sensitizers/oxidizers, as the usage of surplus photosensitizers during the photocatalysis process results in the scavenging effect of the produced oxygen radicals and holes, thereby increasing the time factor for dye degradation/dissipation. Hence, it was inferred that the addition of 2 mM KBrO_3_ as oxidizer/sensitizer, under optimized conditions, i.e., 20 ppm of RB-10 dye, solution pH 2.0, and 50 mg of F127-550 TiO_2_ monolithic photocatalyst, ensured ≥ 98.7% dye dissipation within 15 min, using 150 W/cm^2^ visible light irradiation. Following the photocatalysis process, the photocatalytic efficacy towards the mineralization of RB-10 dye molecules using F108-550, F127-550 and P123-550 TiO_2_ monoliths has been investigated using total organic carbon (TOC) analysis to quantify the remaining organic carbon content after photocatalytic irradiation of RB-10 dye molecules, as shown in Fig. 7h. TOC analysis in the presence of sensitizers, revealed 90% of RB-10 molecules were mineralized within 40 min of photocatalysis using F127-550 TiO_2_ monolith under optimal experimental conditions. Hence, it was inferred that a relatively longer timespan proved imperative for completely mineralizing the degraded pollutant molecules. The superior catalytic performance of the proposed mesoporous TiO_2_-based monolithic photocatalysts has been compared with various literature reports, as tabulated in Table 1^46–67^.

**Table 1:** Comparison of the photocatalytic activity by various TiO_2_-based materials for fabric dye degradation.

S. no.	Photocatalyst	Illumination conditions	Catalyst dosage (mg)	Degradation rate (%)	Pollutant concentration (ppm)	Degradation time (min)	Fabric dye pollutants	Ref
1.	TiO_2_/SiO_2_ Monolith	300 W Xenon Lamp	20	80	6	30	Methylene Blue	^46^
2.	TiO_2_ Nanoparticles	300 W Xenon Lamp	25	96	10	60	Rhodamine B	^47^
3.	TiO_2_ Nanoparticles	300 W Xenon lamp	10	86	10	40	Methylene Blue	^48^
4.	TiO_2_ Nanoparticles	100 W Mercury Vapor Lamp	150	64	10	70	Methylene Blue	^49^
5.	TiO_2_ Nanoparticles	Natural Sunlight	10	62	10	50	Rhodamine B	^50^
6.	(Fe)-doped TiO_2_ Nanoparticles	150 W Philips CFL Bulb	50	93	10	180	Methylene Blue	^51^
7.	Titania Monolith	500 W Hg (Xe) globe	500	55	50	60	Methylene Blue	^52^
8.	TiO_2_ Nanoparticles	UV-A (20 W) Lamp	20	90	50	60	Methylene Blue	^53^
9.	Ag-doped TiO_2_ Nanoparticles	400 W UV Lamp	35	76	10	30	Reactive Blue-160	^54^
10.	TiO_2_	125 W Mercury Vapor lamp	0.05	85	50	240	Methylene Blue	^55^
11.	TiO_2_	UV lamp	2.5	94	6	190	Rhodamine B	^56^
12.	TiO_2_	40 W UV lamp	2	85	10	140	Direct Red 23	^57^
13.	TiO_2_	UV lamp	0.025	82	25	90	Eriochrome Black T	^58^
14.	TiO_2_	UV lamp	0.6	93	50	60	Violet-26	^59^
15.	TiO_2_	40 W blacklight blue fluorescent lamps	1.6	98	45	90	Reactive Red 120	^60^
16.	TiO_2_	Xenon 254-nm lamps	0.09	100	20	120	Reactive Orange 16	^61^
17.	TiO_2_	Natural Sunlight	0.2	100	1.2 × 10^− 4^ M	240	Reactive Yellow 17	^62^
18.	TiO_2_	40 W UV black fluorescent lamps	2	94	100	150	Direct Yellow 12	^63^
19.	TiO_2_	125 W Mercury Vapor lamp	0.3	95	100	180	Reactive Red 198	^64^
20.	TiO_2_	45 W UV lamp	2	91	30	300	Reactive Black 5	^65^
21.	TiO_2_	16 W UV lamp	0.5	96.6	1000	45	Remazol Brown	^66^
22.	TiO_2_	15 W Mercury Vapor lamp	1.75	88	0.2 × 10^− 4^ M	60	Bismarck Brown R	^67^
10.	**Undoped Mesoporous TiO**_**2**_ **Monolith**	**150 W Tungsten Lamp**	**50**	**98.7**	**20**	**15**	**Reactive Brown-10**	**Present Work**

The reusability prospects of the synthesized monolithic photocatalyst constitute a crucial parameter for assessing its practical applicability and suitability for large-scale catalytic processes. A series of six trials were conducted consecutively under optimized experimental conditions to investigate the reusability nature of the F127-550 TiO_2_ monolith for the photocatalytic degradation of persistent organic pollutants, as shown in Fig. 7i. Following each photocatalysis experiment, the monolithic photocatalyst material was recovered from the solution and subjected to a cleaning process involving rinsing with a mixture of deionized water and ethanol to eliminate any residual RB-10 dye adhering to the surface of the photocatalyst. The recovered photocatalyst was vacuum-dried overnight at 40ºC. The plot revealed that even after five cycles, the proposed photocatalyst demonstrated commendable reusability and robust photocatalytic activity in decontaminating the dye molecules. Nevertheless, a slight decline was observed in the dye removal efficiency after five cycles compared to the initial run, which was attributed to the marginal loss of the photocatalyst material during the washing and retrieval process.

Further, we have confirmed the stability of the F-127 550 TiO_2_ monolith photocatalyst through p-XRD and FE-SEM analysis of the reused material. The relevant results have been provided in the Electronic Supplementary Material (Fig. S4a–c). The p-XRD and FE-SEM results showed no significant differences between the fresh and recycled catalysts after six reuse cycles for the photocatalytic degradation studies, demonstrating the prepared photocatalyst’s excellent chemical and thermal stability. Besides, the pollutant molecule might have penetrated deep inside the internal cavities of the photocatalyst, thereby rendering them inaccessible for efficient removal. These factors could be the reason behind the marginal decline in the dye adsorption and degradation efficiency of the F127-550 TiO_2_ monolithic photocatalyst.

Nonetheless, the findings evidenced rapid adsorption kinetics and excellent reversibility/renewable characteristics for the proposed mesoporous monolithic TiO_2_ photocatalyst, thereby underscoring their practical applicability and significance for pollutant dissipation in real-world applications. The degradation pathway of RB-10 molecules using F127-550 TiO_2_ monolith under visible light irradiation was investigated using HR-MS. The analysis identified sixteen intermediates (photoproducts) during the dissipation of RB-10 dye molecules, which were depicted in Scheme S1 & Fig. S5a–c (Electronic Supplementary Material).

### Mechanism of photocatalysis

Based on the material characterization and photocatalysis studies, a potential photocatalytic reaction mechanism and electron transfer processes for the degradation of RB-10 dye by the F127-550 TiO_2_ monolith have been proposed. The valence band energy (E_VB_) values were determined from the VB-XPS analysis, while the conduction band potential (E_CB_) of the semiconductors was calculated using the obtained band gap and valence band using the following empirical Eq. (2)

2 $$\:{E}_{VB}={E}_{CB}+{E}_{g}$$

Here, the term E_VB_ and E_g_ presents the valence band and energy band gap of the TiO_2_ monolithic photocatalyst. The conduction band (CB) and valence band (VB) edges of the F127-550 TiO_2_ monolith were − 0.47 and + 2.43 eV, respectively. For undoped TiO_2_ monoliths, visible light-induced photocatalysis was achieved by electronic structural modifications by inducing lattice/surface defects. The energy band gap narrowing caused by the surface/lattice defects and oxygen vacancies extends the photocatalytic activity to the visible light spectrum. These defect-induced-trapping centers facilitate the effective separation of the photogenerated electrons and holes, promoting e-/h + pair separation and reducing the recombination effect, thereby prolonging the carriers’ lifespan for producing reactive oxygen species as radical intermediates for dye dissipation. The nature and coexistence of these defects are highly dependent on the preparation conditions.

For instance, annealing at temperatures below 450 °C results in only oxygen vacancies, while higher temperatures induce oxygen vacancies and Ti^3+^ interstitial sites. At elevated temperatures above 650℃, the porosity of TiO_2_ decreases, affecting its ability to adsorb reactant molecules for catalysis reactions. It has been suggested that these defects, particularly oxygen vacancies and Ti^3+^ interstitials, play a significant role in the photocatalytic decomposition of RB-10 dye under visible light irradiation. Notably, oxygen vacancies have been found to enhance the decomposition of RB-10 dye positively. Under visible light irradiation, electrons are excited to impurity levels created by Ti^3+^ and oxygen vacancies. Oxygen vacancies in TiO_2_ photocatalysts contribute to excellent photocatalytic activity and improved light absorption. These vacancies are generated on the TiO_2_ surface, where oxygen atoms attach and form hydroxyl groups through O_2_ radicals. In addition, the photoinduced electron lifetime in oxygen vacancy traps is longer than the conduction band, favoring oxygen reduction to produce superoxide radicals and enhancing photocatalytic activity. Further, Ti^3+^ defects within the material bulk narrowed the energy band gap, with Ti^3+^ interstitial sites primarily accounting for visible light absorption. The oxygen vacancies produced by heating TiO_2_ introduce Ti^3+^ interstitials having an extra electron than Ti^4+^. Reduced TiO_2_ has altered band structure with localized states within the energy band gap. Oxygen vacancies and Ti^3+^ states induce highly reactive sites on the monolithic TiO_2_. The defective sites could be promising for various photocatalytic reactions under visible light radiation. However, using dyes for visible-light activity testing may not accurately reflect the proper visible response due to the competitive sensitization mechanism of the dyes.

Visible light-induced photocatalysis for undoped TiO_2_ has been achieved by changing the electronic energy states by lowering the interstitial energy levels through lattice/surface defects and oxygen vacancies. The imperfections caused by oxygen vacancy in TiO_2_ photocatalysts exhibit excellent photocatalytic activity and improved visible light absorption properties. Under visible light irradiation, F127-550 TiO_2_ undergoes a process wherein electrons are directly transferred from the valence band to the conduction band by narrowing the energy band gap with interstitial fermi levels. As the conduction band of titanium dioxide is an electron acceptor, it readily accepts the electrons and forms superoxide radicals (∙O_2_^−^). The photogenerated holes (h^+^) in the valence band and the photoexcited electrons (e^−^) in the conduction band react with the adsorbed water molecules or surface hydroxyl groups (OH^−^) to form hydroxyl radicals (OH∙) that promote the degradation of the pollutant molecules. Consequently, under visible light irradiation, these reactive oxygen radicals play a crucial role in breaking down the adsorbed RB-10 dye molecules on the photocatalyst surface to smaller, less toxic molecules, leading to complete mineralization. The F127-550 TiO_2_ monolith photocatalyst demonstrated outstanding performance in initiating visible light-induced electronic transitions, separating electron-hole pairs, significantly contributing to its exceptional visible light photocatalytic efficacy.

**Fig. 8:**
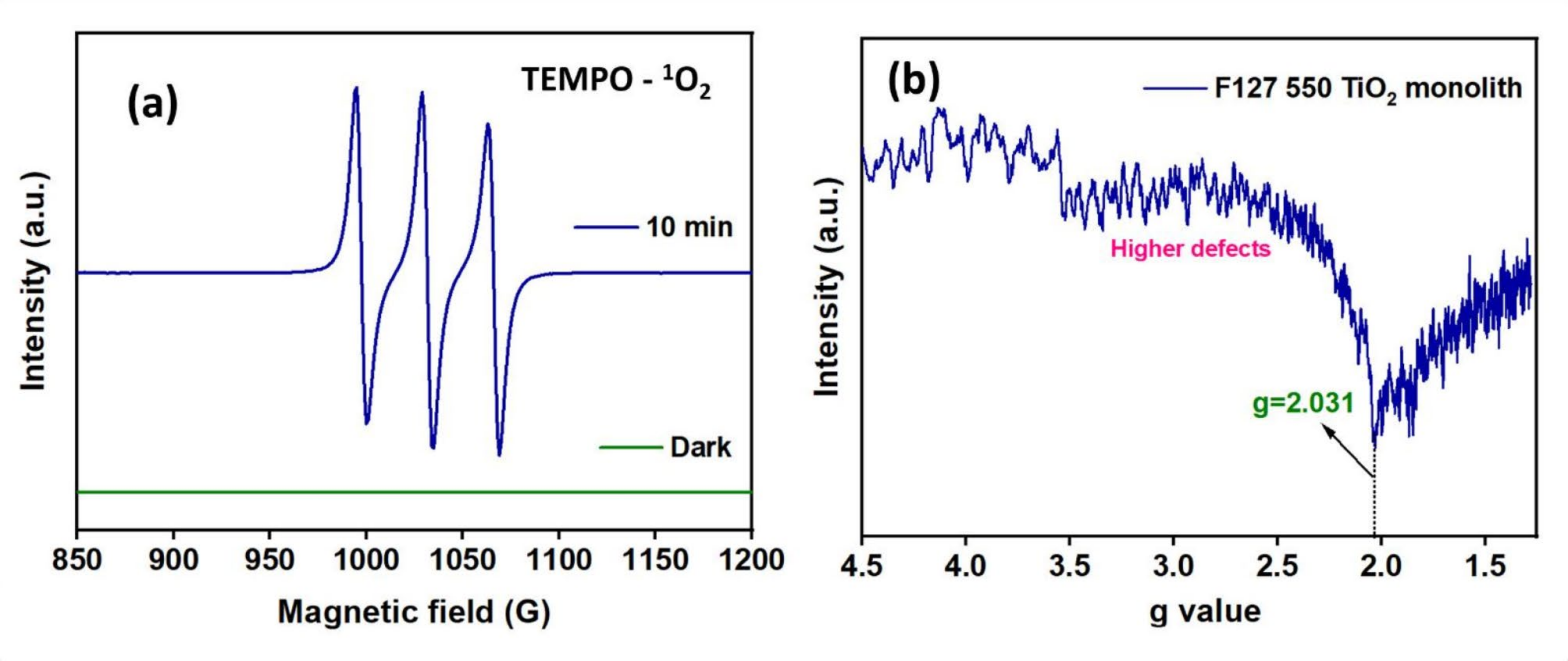
**a** EPR spectra for detecting^1^O_2_ in the presence of TEMPO and **b** EPR spectra confirming oxygen vacancies for F127-550 TiO_2_ monolith.

For understanding the visible light photocatalytic mechanism of undoped TiO_2_ monolithic matrix, electron paramagnetic resonance (EPR) spectroscopy represents a sensitive tool for the direct detection of paramagnetic surface defects in the solid matrix and the quantification of vacancy-induced^1^O_2_ generation to comprehend all the possible imperfection levels that impact on the photocatalytic activity. Figure 8a represents the EPR spectral data measurement at room temperature that reveals three characteristic peaks of TEMPO-^1^O_2_ in the F127-550 TiO_2_ monolith to confirm the presence of singlet oxygen. Figure 8b depicts the EPR spectra for the F127 550 TiO_2_ monolith that exhibits several peaks apart from the prominent peak that indicates the presence of several types of defects. The presence of a signal at g = 2.031 with a broad underline peak for the F127-550 TiO_2_ monolith represents the existence of significant oxygen vacancies. Hence it can be inferred that the electrons trapped at Ti^4+^ sites can form Ti^3+^ and holes at subsurface oxide ions that can form O^−^. The g-value can be assigned to the surface Ti^3+^ (i.e., electron-trapped due to Ti^3+^ at the surface of the TiO_2_). Combining singlet oxygen and Ti^3+^ defects can significantly enhance the performance of the F127-550 TiO_2_ monolith, making it more effective for the degradation of RB-10 dye pollutants. In an aqueous medium, active components such as hydroxyl radical (∙OH), holes (h^+^), and electrons (e^-^) can play significant roles in the photodegradation of organic pollutants. Hence, to gain insight into the underlying mechanism and the reactive species of photocatalytic degradation of RB-10 on the F127-550 TiO_2_ monolith, experiments were carried out using various radical quenchers, as discussed in the Electronic Supplementary Material (Fig. S6). The trapping experiments have been carried out using radical quenchers such as isopropyl alcohol (IPA), disodium ethylenediaminetetraacetate (EDTA-2Na^+^) and p-benzoquinone (p-BQ) for ∙OH, h^+^ and O_2_^-^∙, respectively.

## Discussion

In this study, a simple strategy for the successful synthesis of undoped mesoporous TiO_2_ monoliths using different triblock copolymers, such as F108, P123, and P127, has been adopted for the efficient visible-light-induced photocatalytic degradation of RB-10 dye pollutant. The interaction between the dye molecules and photocatalyst, particularly in aqueous media, determines which of the surfactants, F127, P123, or F108, is most suitable for RB-10 degradation. The use of Pluronic F127 significantly enhanced the photocatalytic degradation rate by modifying the morphology, particle size, and crystallite size and introducing defect energy levels that altered the photocatalyst’s energy band, thereby influencing the light absorption and charge carrier separation phenomena, as confirmed by DRS, PLS and XPS data. The resulting TiO_2_ exhibited a dual anatase-rutile crystalline phase with Pluronic F127, whereas no distinct dual-phase crystalline structure was formed with other surfactants such as F108 and P123. TiO_2_ prepared with surfactants demonstrated superior photocatalytic performance compared to commercial TiO_2_, attributed to increased surface area and smaller pore and crystallite sizes.

The mesoporous TiO_2_ monolithic photocatalysts were prepared under different calcination temperatures (450 °C, 550 °C and 650 °C). TiO_2_ monoliths synthesized using Pluronic F127 (SDA) and calcinated at 550 °C showed a lower energy band gap and enhanced visible light absorption properties. At 450℃ calcination, the TiO_2_ crystal lattice revealed only oxygen vacancy, but at 550℃, the coexistence of oxygen vacancies and Ti^3+^ interstitials was prominent, thus narrowing the energy band gap and increasing the formation/separation of electron-hole pairs for enhanced photocatalytic performance. The crystallite size and band gap were the desired characteristics of the photocatalysts that influence their photocatalytic activity. The oxygen vacancies formed due to their high-temperature treatment also affected the band gap. As the crystallite size increases, it decreases the band gap of the sample through size quantization. A red shift in the energy band gap with increasing calcination temperature has been attributed to the quantum confinement effect. Smaller crystallite sizes generally increase the surface area, an advantageous feature for photocatalysis as it offers voluminous active sites for adsorption and photocatalytic reactions, potentially enhancing degradation efficiency. However, tiny crystallites can accelerate the charge-charge recombination effect, reducing the photocatalytic performance. Therefore, the optimal crystallite size was essential to balance these effects, as in the F127-550 TiO_2_ monolith, for significantly enhancing the dissipation of RB-10 dye molecules. The visible light-induced photocatalysis of the optimized F127-550 TiO_2_ monolith was evaluated using RB-10 fabric dye.

Compared to the anatase or rutile phase of TiO_2_, the heterophase (anatase-rutile)TiO_2_ monolith has a narrow energy band gap suitable for ample visible light absorption. The band gap of the F127-550 TiO_2_ monolith was 2.92 eV, where anatase and rutile phases coexisted at a calcination temperature of 550℃. Maintaining an ambient energy band gap remained crucial, as an overly narrow gap can reduce photocatalytic efficiency due to smaller redox potential and a more significant charge carrier recombination. It was evident that the alterations in chemical composition, pore size distribution, specific surface area and band gap energy ultimately resulted in better photocatalytic performance. Based on the experimental parameters, faster dye degradation was achieved within 15 min of irradiation at pH 2.0 for 20 ppm of RB-10 dye solution, using 50 mg of photocatalyst and 2 mM KBrO_3_. The F127-550 TiO_2_ monoliths exhibited robust stability and can be retrieved/reutilized for several consecutive cycles. Hence, the as-prepared F127-550 TiO_2_ monoliths offer great potential as a hyperactive catalyst by efficiently harvesting visible light energy in the environmental decontamination of persistent organic pollutants. Moreover, the high stability/durability of the F127-550 TiO_2_ monolithic photocatalyst serves as an additional advantage that can prove handy during real-time practical conditions.

## Methods

### Chemicals and reagents

Titanium(IV) isopropoxide (TTIP), Pluronic P123 (EO_20_PO_70_EO_20_), F108 (EO_141_PO_44_EO_141_), Pluronic F127 (EO_100_PO_65_EO_100_), 1,3,5-trimethyl benzene (C_6_H_3_(CH_3_)_3_, or TMB) were purchased from Sigma-Aldrich. The pH adjustments were performed using 0.2 M buffer of ClCH_2_COOH-CH_3_COOH (pH 1–3), CH_3_COONa-CH_3_COOH (pH 4–6), CH_3_CO_2_NH_4_-NH_3_ (pH 7–8) and NH_4_Cl-NH_3_ (pH 9–10). The diazo-based textile fabric dye, Reactive Brown 10 (RB-10), was procured from Sigma-Aldrich.

### Instruments for material characterization

A detailed description of the instruments used to characterize the synthesized monolithic photocatalyst materials has been provided in the Electronic Supplementary Material (ESM).

### Mesoporous TiO_2_ monoliths composition

The mesoporous TiO_2_ monoliths were synthesized through an instant direct templating liquid-crystal phased approach using various tri-block copolymers, i.e., Pluronic F108, P123 and P127 as SDAs. For the TiO_2_ monolith synthesis using F108, 1.6 g of Pluronic F108, 2.0 g of TTIP, and 0.4 g of TMB were mixed and placed in a water bath maintained at 60 °C for 5 min to obtain a homogeneous solution. Then, 1.0 g of H_2_O/HCl (pH 1.3) mixture was added quickly to the above solution, leading to gelation, with a mass ratio of 1.6:0.4:2.0:1.0 for F108:TMB: TTIP: H_2_O/HCl. The translucent gel was aged for 24 h at 40 °C and subsequently at room temperature for 6 h. The aged gel was set to temperature-controlled calcination at 550 °C for 8 h to form the mesoporous TiO_2_ monolith. A schematic representation of the synthesis of mesopore TiO_2_ monoliths has been provided in the Electronic Supplementary Material (Scheme S2). In this line, mesoporous TiO_2_ monoliths were synthesized using P123 and F127 tri-block copolymers as SDAs and were calcined at different temperatures using a similar procedure. Henceforth, TiO_2_ monoliths prepared with F108 SDA, calcined at 450 °C, 550 °C and 650 °C will be denoted as F108-450, F108-550 and F108-650, respectively. In this line, for P123 and F127 SDA, based TiO_2_ monoliths at different calcinated temperatures, i.e., 450, 550 and 650ºC, are denoted as P123-450, P123-550, P123-650 and F127-450, F127-550 and F127-650, respectively.

### Photocatalytic studies

The synthesized mesoporous TiO_2_ monoliths were tested for their photocatalytic activities using RB-10 textile dye solution of varying concentrations to examine the photocatalytic properties of the undoped TiO_2_ monoliths. Photocatalytic reactions are carried out under UV/Visible light, and the photocatalytic degradation of RB-10 dye was significant under the visible light region, using mesoporous TiO_2_ monoliths from different SDAs at 550 °C and 650 °C. Likewise, a mere dispersion of photocatalyst in the dye solution (in the absence of light) showed no significant decrease in the RB-10 dye concentration. Based on these findings, it was inferred that for the complete dissipation of RB-10 molecules, a synergetic combination of photocatalyst and illumination remained imperative. The extent of photocatalytic degradation of RB-10 solution was monitored using UV-Vis spectrophotometry. The visible light (tungsten filament lamp) source with 150 W/cm^2^ intensity was used to conduct the photocatalytic dye degradation experiments (in a 100 mL capacity quartz/glass tube) utilizing a double-walled water-cooled annular type photoreactor to host the visible light lamp and a series of quartz/glass tubes containing RB-10 dye solutions. The annular photoreactor was specially designed with in-built cooling fans, light reflectors and a water-circulation unit. For photocatalysis studies, an optimal amount of photocatalyst was dispersed in 50 mL of appropriate concentrations of RB-10 dye solution, maintained at a specific pH condition. For efficient degradation of RB-10 dye, various dye concentrations (10–50 ppm) and photocatalyst dosages were investigated at different light intensities.

The effect of various experimental physio-chemical parameters, including solution pH, nature/amount of TiO_2_ monolith (based on SDA and temperature), degradation kinetics, and the role of sensitizer/oxidizers, were comprehensively analyzed to study the photocatalytic degradation efficiency of RB-10 dye solution. The maximum absorption wavelength (λ_max_) of 526 nm was observed for RB-10 to monitor the degradation process using a UV-Vis spectrophotometer. The mass balance was maintained by withdrawing only small volumes of sample solutions at specific intervals to analyze the extent of dye degradation/dissipation and mineralization.

## Electronic supplementary material

Below is the link to the electronic supplementary material.

Supplementary Material 1

## Data Availability

The authors declare that the data supporting the findings of this study are available within the paper and its Supplementary Information files.
