# Supplementary material for: Visible light responsive heterophase Titania monoliths for the fast and efficient photocatalytic decontamination of organic pollutants
Source: Sci Rep. 2024 Nov 10;14:27441. doi: 10.1038/s41598-024-79285-3 (PMC11551148; doi:10.1038/s41598-024-79285-3)
Supplement: Supplementary file 1 — Supplementary Material 1 [file 41598_2024_79285_MOESM1_ESM.docx]

**Visible Light Responsive Heterophase Titania Monoliths for the Fast and Efficient Photocatalytic Decontamination of Organic Pollutants**

Denna Babu ^a,^**^†^**; Dhivya Jagadeesan ^a,^**^†^**; Thejaswini T V L ^a,^**^†^**; Akhila Maheswari Mohan ^a^; Prabhakaran Deivasigamani ^a,^*

^a^ *Department of Chemistry, School of Advanced Sciences, Vellore Institute of Technology (VIT), Vellore, Tamil Nadu 632014, INDIA.*

_______________________________________________________________________________________

**ELECTRONIC SUPPLEMENTARY MATERIAL**

**S1. Material Characterization:**

The phase purity and crystal structure of the mesoporous TiO_2_ monoliths were analyzed through powder X-ray diffraction (p-XRD) using a Bruker D8 Advance X-ray diffractometer. The obtained diffraction peaks confirmed the crystallinity of the samples, and the phase compositions have been confirmed through cross-confirmation with ICDD (standard files). To investigate the surface morphology and the structural pattern of the materials (FE-SEM-EDAX), Hitachi model S-4800 is used. The Seiko TG/DTA 7200 instrument has been used to carry out the thermogravimetric analysis (TGA) by heating the sample at the rate of 15°C/min/N_2_ flow using Pt and Pt-Rh (15%) differential thermocouple. An X-ray photoelectron spectrometer (XPS; Axis 165 model) was employed to investigate the monolithic photocatalyst's chemical state and surface composition. The microstructure and the distribution of elements were studied by HR-TEM-SAED (Tecnai, G_2_ 20 S-Twin) analysis. The band gap measurements were carried out using a Perkin-Elmer lambda 35 model UV-Vis Diffuse Reflectance Spectrophotometer (UV-Vis-DRS). The Micromeritics ASAP 2020 instrument has been used to measure the N_2_ adsorption-desorption (BET) isotherms. The Cary-eclipse fluorescence spectrophotometer has been used to analyze Photoluminescence spectral (PLS) studies. For the photocatalytic degradation of RB-10 dye solution, an annular photoreactor (model Heber HML-LP-MP812) fitted with a 150W/cm^2^ tungsten filament was used as a visible light lamp source. TOC (Total organic carbon) analysis has been carried out using the Elemental Vario model TOC analyzer. For studying the defect sites in the undoped TiO_2_ monolith, a Bruker EMX plus electron paramagnetic resonance (EPR) spectrometer with a microwave frequency of 9.8 GHz was deployed. The mass fragments (photoproducts) of RB-10 were analyzed using a high-resolution mass spectrometer (HR-MS) (model Waters Xevo G2-XS QToF).

**S2. p-XRD Data:**

**Table S1.** Comparison of d-spacing values of TiO_2_ monoliths for SDAs at 550°C.

| **F127-550** | **λ (nm)** | **Miller Indices** | | | **Bragg Angle** | | **d Spacing (Å)** | **d (nm)** |
| --- | --- | --- | --- | --- | --- | --- | --- | --- |
|  |  | **h** | **k** | **l** | **2θ** | **θ** | **d_hkl_ =λ/2sinθ** |  |
|  | 1.5406 | 1 | 0 | 1 | 25.3 | 12.7 | 3.517 | 0.352 |
|  |  | 1 | 1 | 0 | 27.6 | 13.8 | 3.229 | 0.323 |
|  |  | 2 | 0 | 0 | 36.2 | 18.1 | 2.476 | 0.248 |
|  |  | 1 | 0 | 5 | 48.2 | 24.1 | 1.886 | 0.189 |
|  |  | 2 | 1 | 1 | 54.4 | 27.2 | 1.685 | 0.169 |
|  |  | 2 | 0 | 4 | 62.8 | 31.4 | 1.478 | 0.148 |
|  |  |  |  |  |  |  |  |  |
| **P123-550** | **λ (nm)** | **Miller Indices** | | | **Bragg Angle** | | **d Spacing (Å)** | **d (nm)** |
|  | 1.5406 | 1 | 0 | 1 | 25.6 | 12.8 | 3.474 | 0.347 |
|  |  | 0 | 0 | 4 | 38.2 | 19.1 | 2.357 | 0.236 |
|  |  | 2 | 0 | 0 | 48.2 | 24.1 | 1.886 | 0.189 |
|  |  | 1 | 0 | 5 | 54.2 | 27.1 | 1.691 | 0.169 |
|  |  | 2 | 1 | 1 | 63.1 | 31.6 | 1.472 | 0.147 |
|  |  | 2 | 0 | 4 | 75.6 | 37.8 | 1.257 | 0.126 |
|  |  |  |  |  |  |  |  |  |
| **F108-550** | **λ (nm)** | **Miller Indices** | | | **Bragg Angle** | | **d Spacing (Å)** | **d (nm)** |
|  | 1.5406 | 1 | 0 | 1 | 25.6 | 12.8 | 3.473 | 0.347 |
|  |  | 1 | 1 | 0 | 27.8 | 13.9 | 3.211 | 0.321 |
|  |  | 2 | 0 | 0 | 36.3 | 18.2 | 2.470 | 0.247 |
|  |  | 1 | 0 | 5 | 48.4 | 24.2 | 1.880 | 0.188 |
|  |  | 2 | 1 | 1 | 54.7 | 27.4 | 1.677 | 0.168 |
|  |  | 2 | 0 | 4 | 63.5 | 31.8 | 1.464 | 0.146 |

**S3. UV-Vis-DRS and PLS Plot:**

**Table S2.** Energy bandgap data of TiO_2_ monoliths from different SDAs at different calcination temperatures.

| **SDA and temperature-based TiO_2_ Monolith** | **Energy Bandgap (E_g_) (eV)** |
| --- | --- |
| F108-450 | 3.15 |
| P123-450 | 3.17 |
| F127-450 | 3.14 |
| F108-550 | 2.88 |
| P123-550 | 2.90 |
| F127-550 | 2.92 |
| F108-650 | 2.85 |
| P123-650 | 2.81 |
| F127-650 | 2.86 |


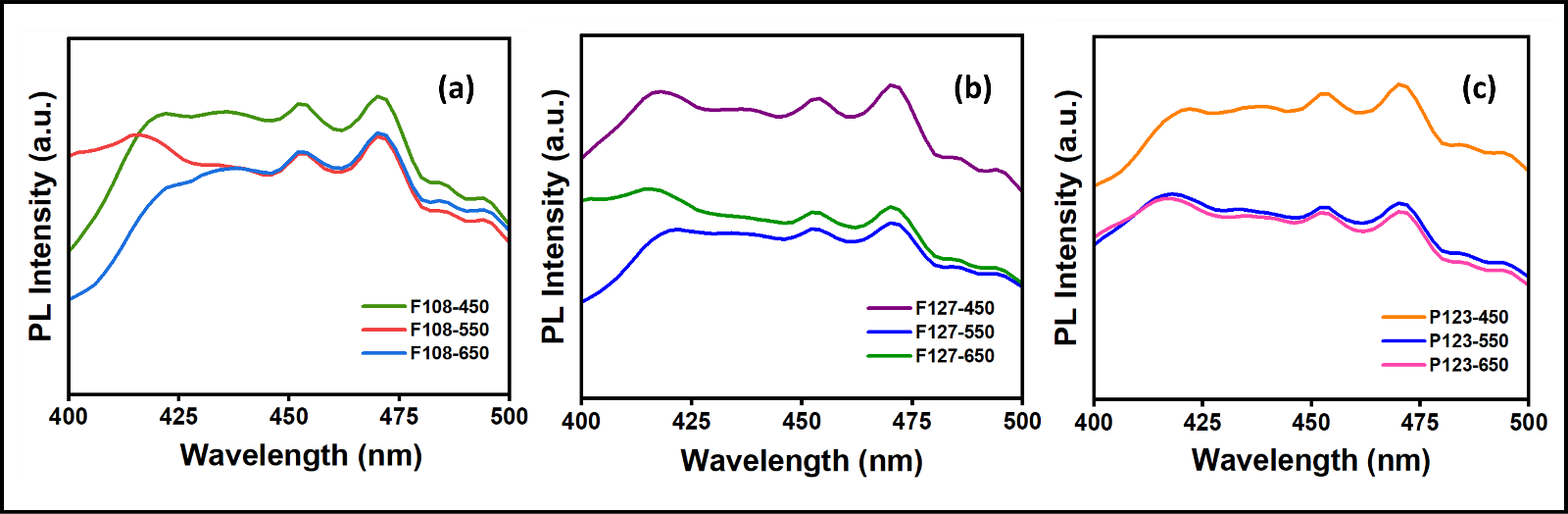


**Fig. S1.** **(a-c)** PLS data for F108, F127 and P123 SDAs-based mesoporous TiO_2_ monoliths calcinated at 450°C, 550°C and 650°C.

**S4. BET/BJH Data:**

**Table S3.** BET surface area and BJH pore size of TiO_2_ monoliths from different SDAs and temperatures.

| **Nature of TiO_2_ Monolith** | **Surface Area (S_BET_), m^2^/g** | **Pore Volume (V_P_), cm^3^/g** | **Pore Diameter (D), nm** |
| --- | --- | --- | --- |
| F127-450 | 112.1 | 0.697 | 5.87 |
| F108-450 | 46.5 | 0.225 | 9.81 |
| P123-450 | 42.2 | 0.181 | 2.62 |
| F127-550 | 105.7 | 0.486 | 4.83 |
| F108-550 | 63.6 | 0.177 | 6.88 |
| P123-550 | 64.3 | 0.103 | 2.85 |
| F127-650 | 52.1 | 0.159 | 5.31 |
| F108-650 | 60.5 | 0.086 | 11.53 |
| P123-650 | 50.3 | 0.070 | 9.24 |

**S5. TG Analysis:**

The TGA pattern for TiO_2_ monoliths synthesized using F127 SDA at different calcination temperatures from 450^-^650°C has been depicted in **Fig S2**. From the TGA plot, a weight loss of 3.3% has been noticed by the F127-450 monolithic sample from 10-450°C. A weight loss of 2.0% is observed within 110°C for the presence of H_2_O molecules. Of the total weight loss (3.3) kept, the remaining 1.3% may remain due to organic precursors and SDA during the synthesis of monoliths. It may also be due to the formation of any possible intermediates during the calcination process.

Nevertheless, beyond 450ºC, no weight loss (0.7%) is observed, and the graphical curve becomes linear. For F127-550 and F127-650 TiO_2_ monolith samples, only 2.3% of weight loss and 2.0% is observed until 450^o^C. However, within 110°C, only 0.5% and 0.3% weight loss was observed for F108-550 and F108-650, respectively, due to the percolated water molecules. Interestingly, it is noticed that the hydrophilicity of the F108-450 is more than F127-550 and F127-650 samples due to the higher percentage of weight loss that has been observed within 110°C. In general, the TiO_2_ monoliths prepared from F127 SDA might exhibit better photocatalytic activity due to their enhanced hydrophilic character that leads to increased water percolation through the monolith's mesoporous channels, thereby leading to significant interaction with the monolithic surface followed by dye dissipation upon light irradiation.


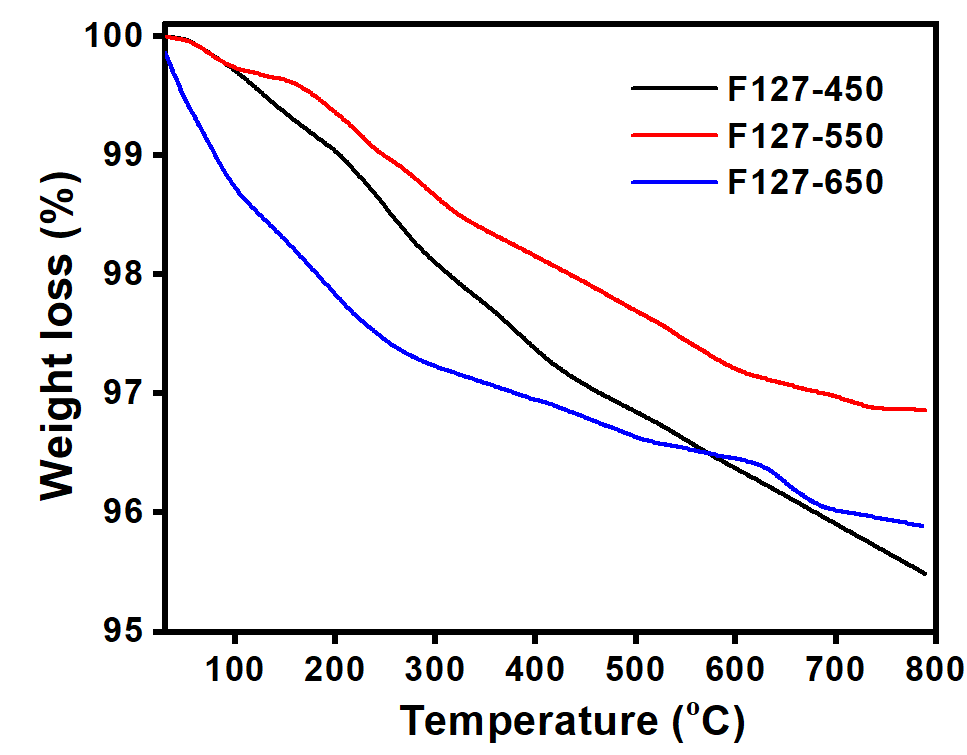


**Fig. S2.** TGA plot of F127 SDA-based mesopore TiO_2_ monolith prepared at different calcination temperatures.

**S6. XPS Analysis:**

The high-resolution deconvoluted spectra for the Ti*2p* orbital state present three peaks in the binding energy range of 457-468 eV for P123, F127 and F108 SDA-based TiO_2_ monoliths at 550°C, as shown in **Fig S3(a)**. The two deconvoluted components located at 459.3 and 464.9 eV for F108-550, 459.5 and 465.1 eV for F127-550 and 459.1, 464.9 for P123-550 were assigned to Ti*2p_3/2_* and Ti*2p_1/2_* orbital states indicating the existence of +4 oxidation state of Ti. The peaks at the binding energy region of 459.3, 459.6 and 459.4 eV correspond to the Ti*2p_1/2_* in +3 oxidation state of Ti_2_O_3_ for F108-550, F127-550, and P123-550, respectively. The shift in the Ti*2p* peak suggests the presence of lattice defect-induced Ti^3+^ that contributes to the bandgap narrowing. The photocatalytic behavior of TiO_2_ was enhanced by the presence of Ti^3+^ defects combined with the lattice distortion. In this line, the high-resolution deconvoluted O*1s* spectra of TiO_2_ monoliths derived from F108, F127 and P123 SDAs at 550°C have been depicted in **Fig S3(b)**. The deconvoluted O*1s* spectra from P123-550, F127-550 and F108-550 TiO_2_ monolith revealed binding energy peaks at 531.0, 531.7, and 531.1 eV corresponding to the lattice oxygen in TiO_2_ monolith. Similarly, the binding energy peaks at 530.4, 530.7, and 530.5 eV correspond to the oxygen species of Ti_2_O_3,_ and the binding energy peaks at 529.9, 530.3 and 530.1 eV belong to the surface adsorbed hydroxyl oxygen (OH) group of TiO_2_ monoliths derived from F108-550, F127-550, and P123-550, respectively.


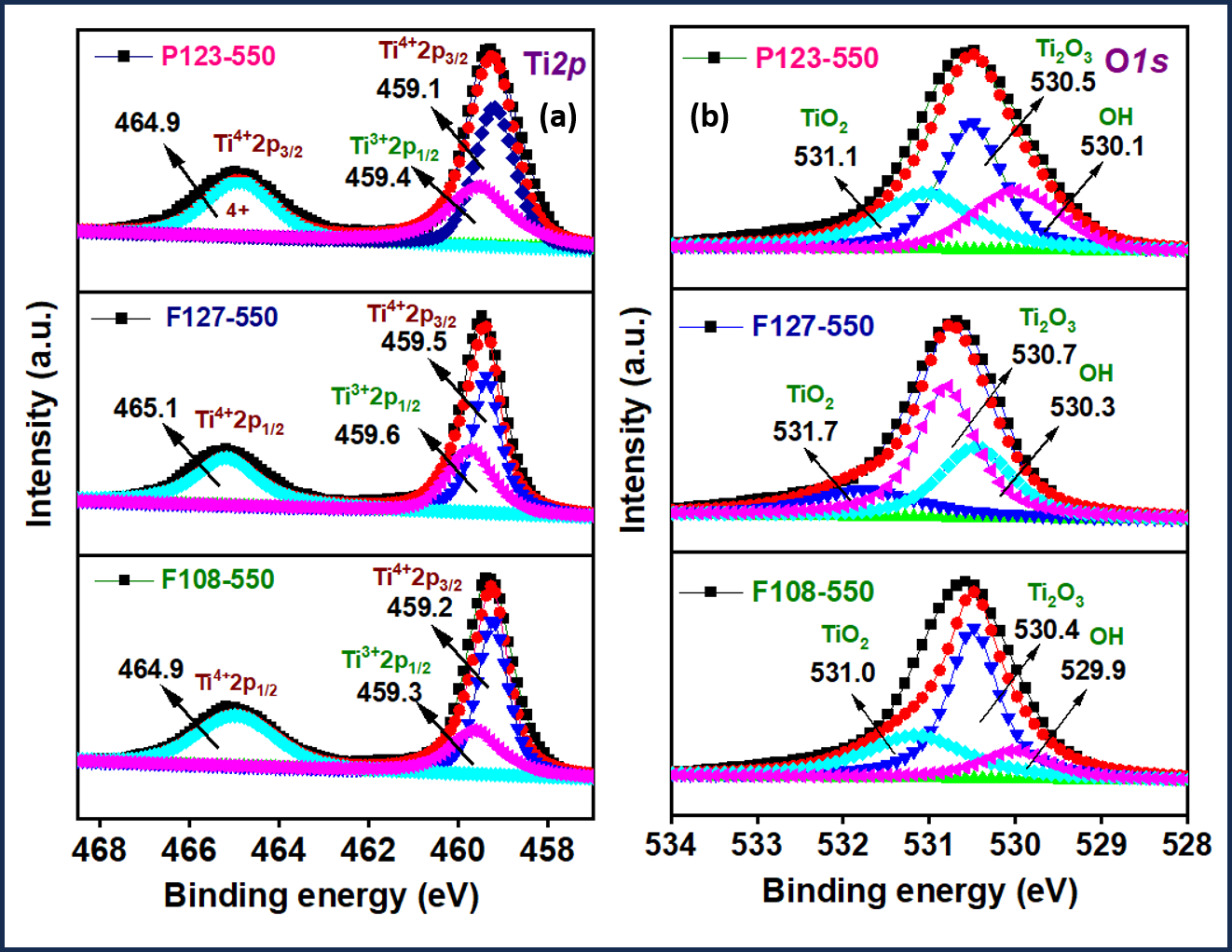


**Fig. S3.** High-resolution deconvoluted XPS data for **(a)** Ti*2p* and (**b)** O*1s* orbital states, respectively, of TiO_2_ monoliths derived from F108, F127 and P123 SDAs at 550°C.

**S7. Characterization of Reused Photocatalyst:**

**
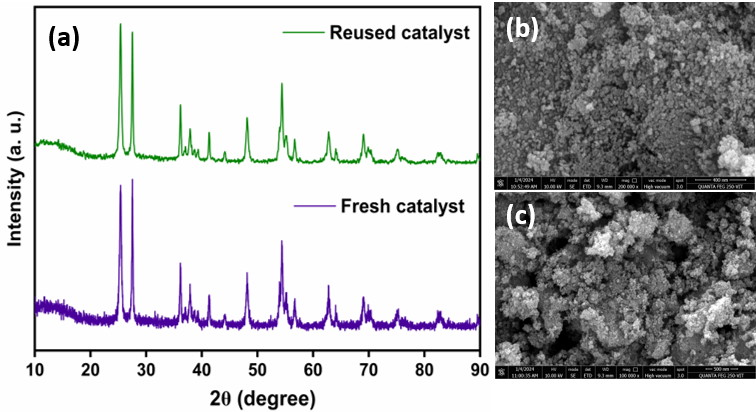
**

**Fig. S4. (a)** p-XRD of fresh and reused F127-550 TiO_2_ monolith and **(b-c)** FE-SEM images of reused F127-550 TiO_2_ monolith.

**S8. HRMS Analysis:**


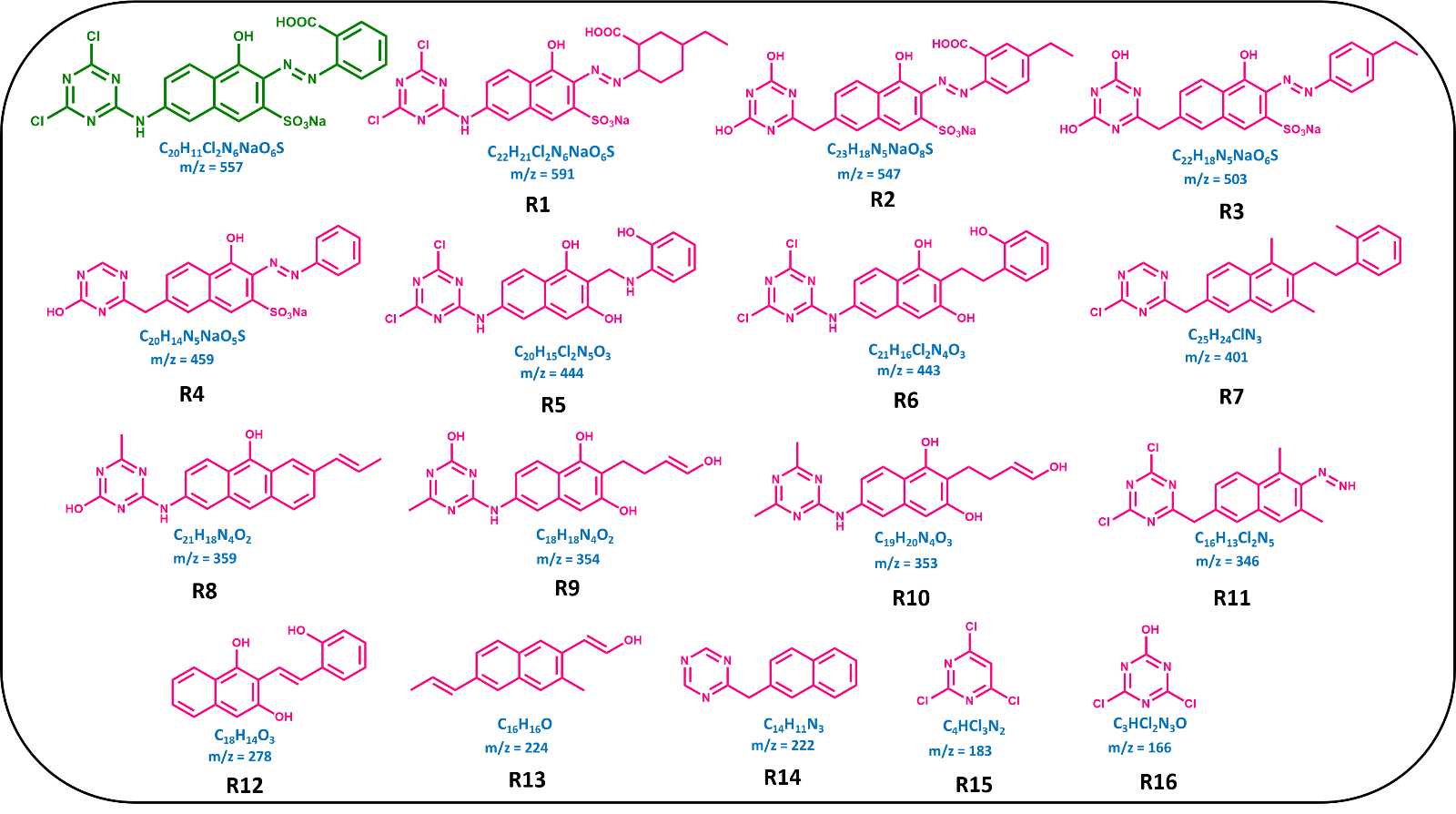


**Scheme S1.** Photoproducts of RB-10 derived using F127-550 TiO_2_ monolith photocatalyst from the visible-light-induced photocatalytic degradation.


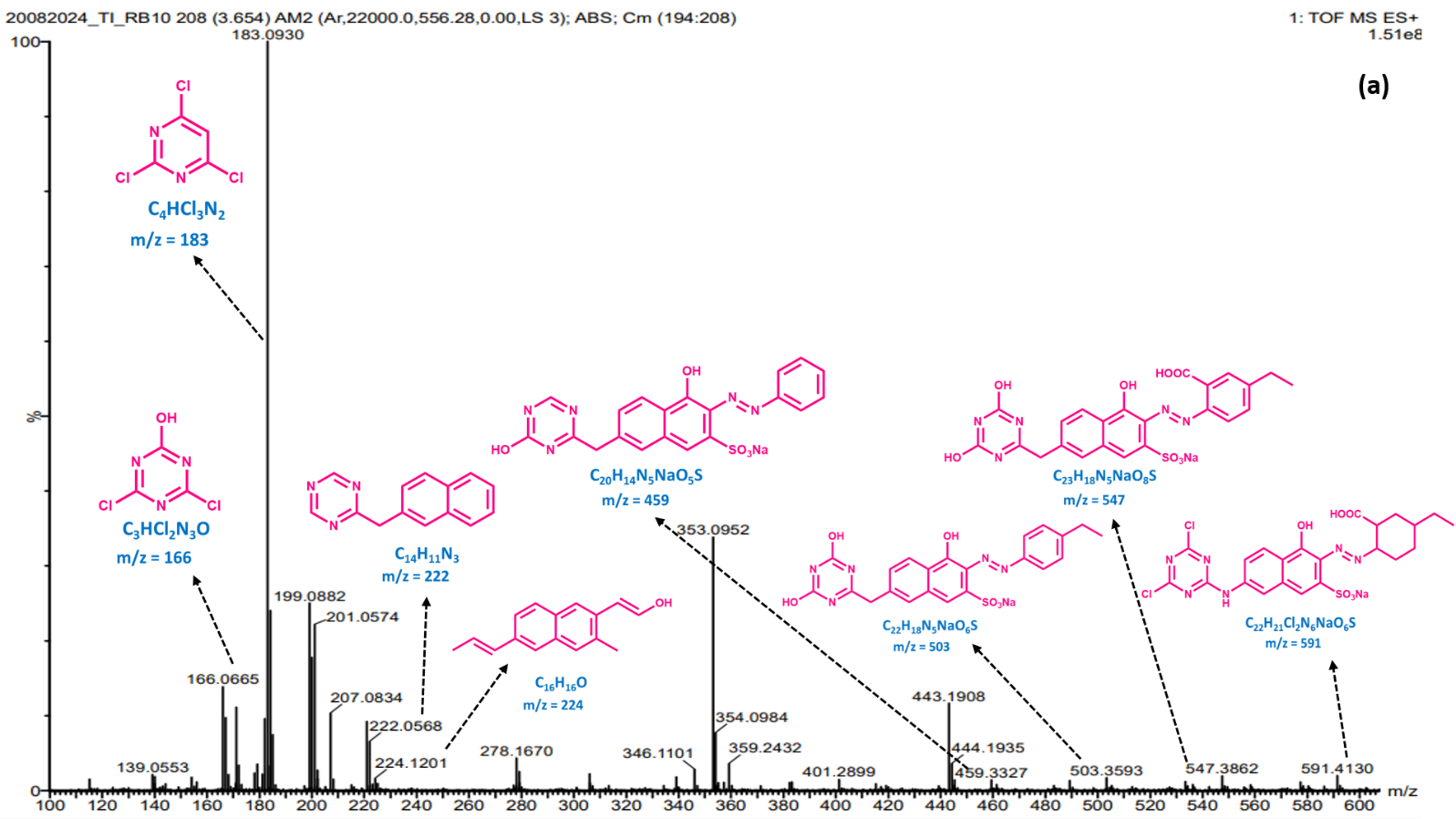


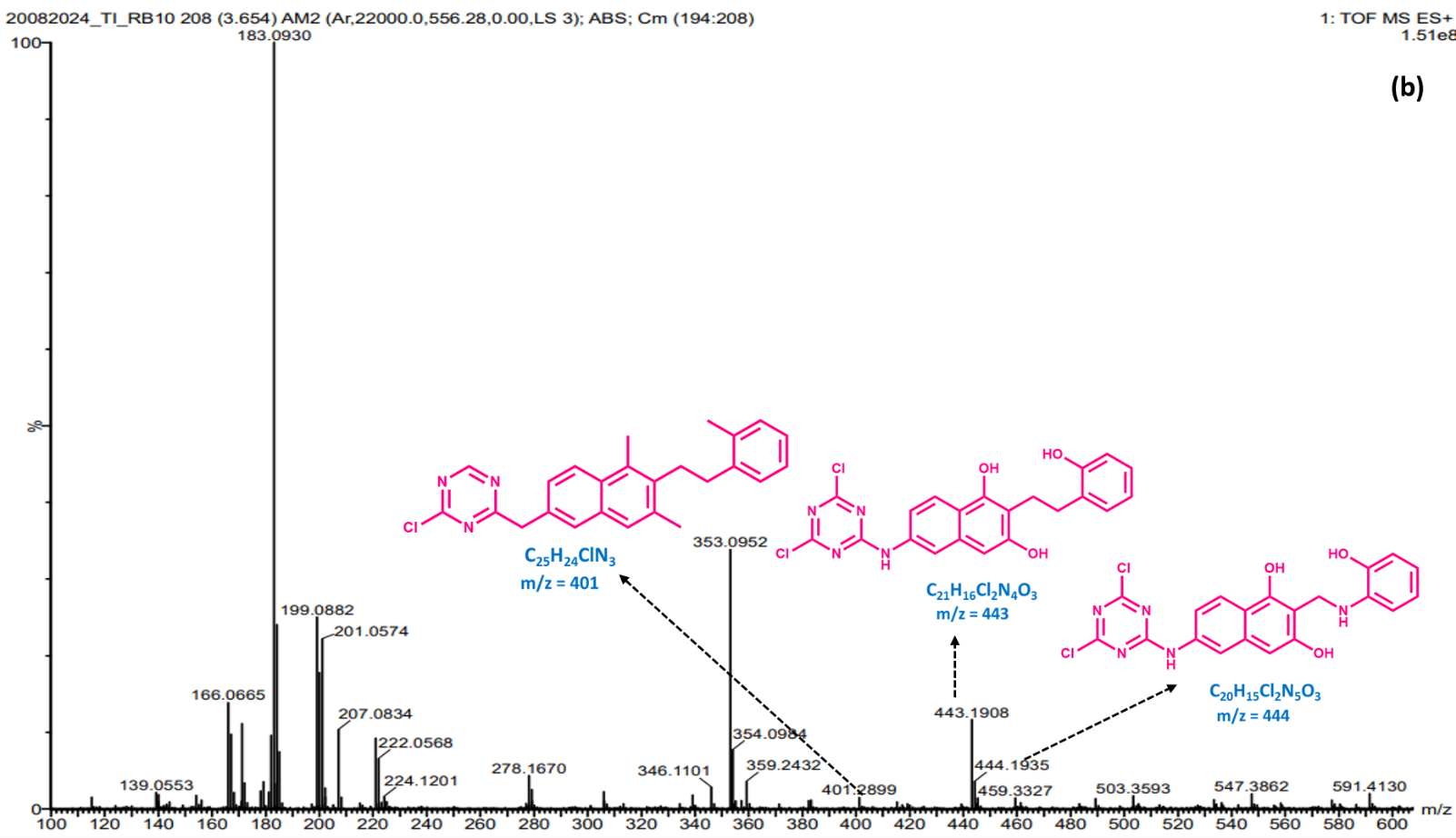


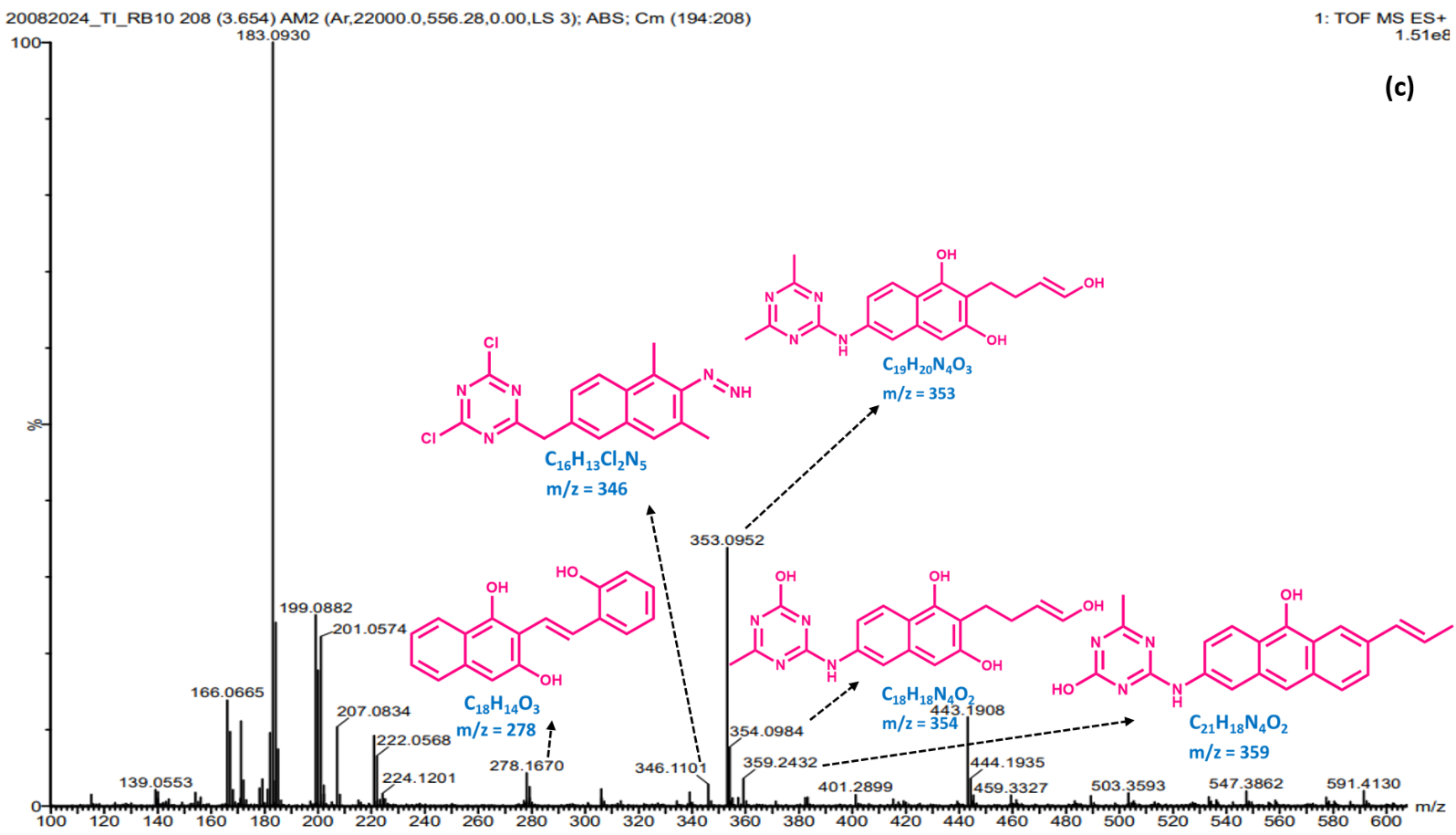


**Fig. S5. (a-c)** HR-MS analysis of RB-10 photoproducts generated under photocatalytic visible light irradiation in the presence of F127-550 TiO_2_ monolith.

**S9. Role of Radical Scavengers:**

Various scavengers were employed to gain further insight into the underlying mechanism and the reactive species of photocatalytic degradation of RB-10 on the F127-550 TiO_2_ monolith. In an aqueous medium, active components such as hydroxyl radical (∙OH), holes (h^+^), and electrons (e^-^) can play significant roles in the photodegradation of organic pollutants. The trapping experiments have been carried out using radical quenchers such as isopropyl alcohol (IPA), disodium ethylenediaminetetraacetate (EDTA-2Na^+^) and p-benzoquinone (p-BQ) for ∙OH, h^+^ and O_2_^-^∙, respectively.

As presented in **Fig. S6**, according to the results in this system, the test without scavenger shows that the photocatalytic degradation of RB-10 dye over F127-550 Ti monolith was 95.3% after 15 min visible light irradiation. When IPA was added, no significant effect on the degradation of RB-10 was observed, which subsequently concluded ∙OH radical did not play any significant role as the active species. While p-BQ and EDTA severely inhibited the degradation of RB-10 dye, indicating that (·O_2_^–^) and (h^+^) were the main active substances leading to RB-10 degradation under visible light irradiation. More specifically, the following order was obtained using various scavengers: no scavenger (95.3%) > IPA (80.5%) > EDTA-2Na^+^ (75.2%) > p-BQ (35.5%) for 15 min visible light irradiation. Based on these results, it could be argued that superoxide radicals (·O_2_^–^) play a vital role in the photodegradation reaction, followed by holes (h^+^), whereas the contribution of hydroxyl radical (·OH) could be considered minor.

**

**

**Fig. S6.** Scavengers test using F127-550 Ti monolith

**S10. Schematic Representation of the TiO_2_ Monolith Synthesis:**


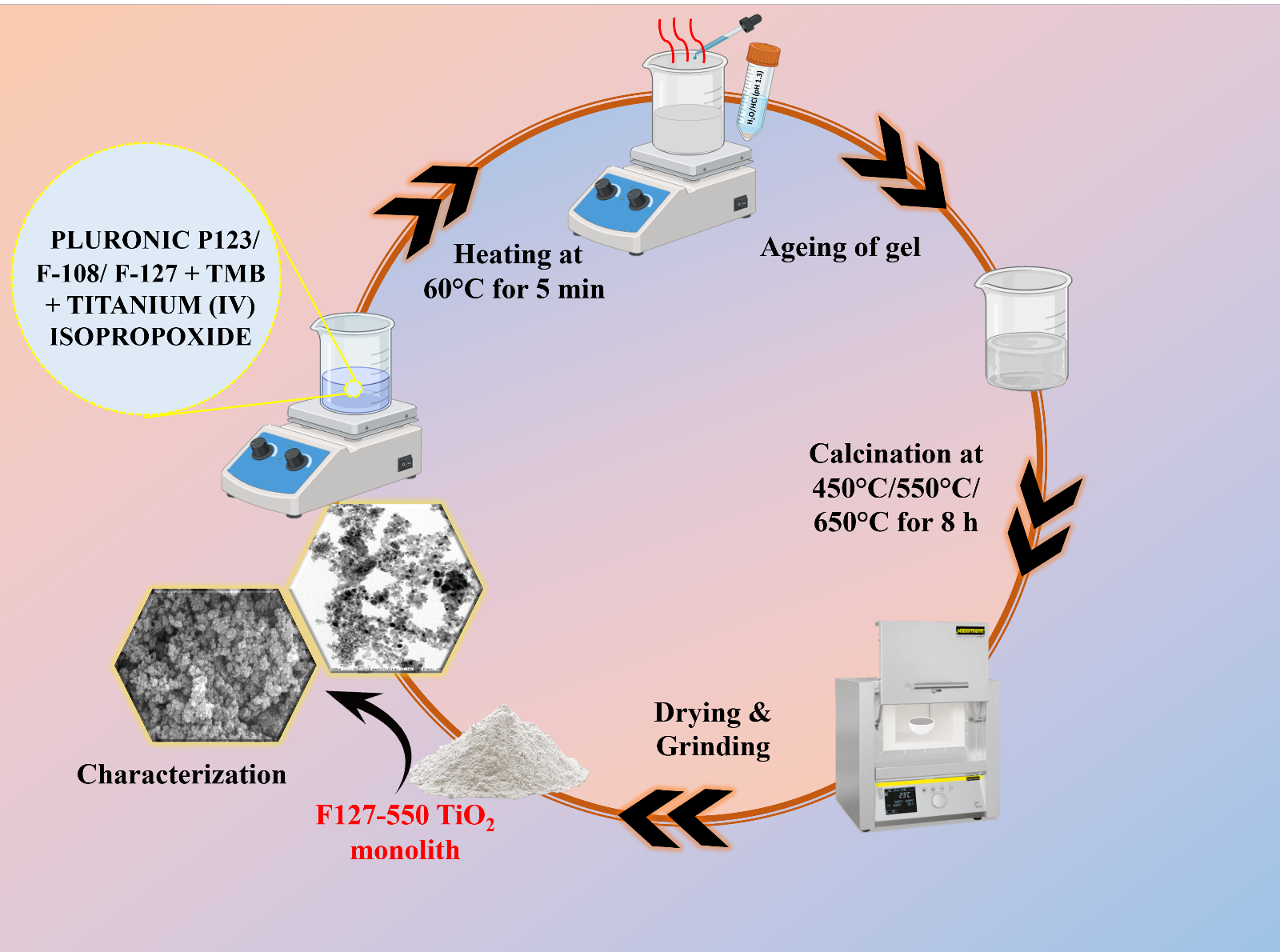


**Scheme S2.** Schematic representation of the sequences related to the synthesis of mesoporous TiO_2_ monolith.
